# Celastrol Inhibits Porcine Epidemic Diarrhea Virus Replication by Promoting ROS‐Mediated Apoptosis

**DOI:** 10.1155/tbed/4020619

**Published:** 2025-12-12

**Authors:** Junhai Zhu, Kaifang Yang, Pengfei Zhang, Hao Yuan, Nan Yan, Longxiang Zhang, Meiyu Jia, Yue Wang

**Affiliations:** ^1^ College of Veterinary Medicine, Southwest University, No 2, Tiansheng Street, Chong Qing, 400715, China, swu.edu.cn

**Keywords:** antiviral drug, apoptosis, celastrol, network pharmacology, porcine epidemic diarrhea virus

## Abstract

Porcine epidemic diarrhea virus (PEDV) causes acute, highly contagious enteric disease in pigs, leading to severe economic losses, particularly due to high mortality in suckling piglets. Currently, no specific antiviral drugs are available. In this study, we evaluated the anti‐PEDV potential of celastrol, a natural triterpenoid derived from *Tripterygium wilfordii*, in Vero E6 cells. We found that celastrol significantly inhibited PEDV replication in a dose‐dependent manner, primarily targeting the postentry stage of the viral life cycle. Network pharmacology analysis highlighted apoptosis as a key signaling pathway, and mechanistic studies revealed that celastrol enhanced PEDV‐induced reactive oxygen species (ROS) accumulation, which triggered apoptosis and suppressed viral RNA synthesis, protein expression, and progeny production. Importantly, inhibition of ROS abolished celastrol’s antiviral activity, confirming a ROS‐dependent mechanism. Furthermore, celastrol demonstrated inhibitory effects against porcine deltacoronavirus (PDCoV) and porcine reproductive and respiratory syndrome virus (PRRSV) in vitro. These findings suggest celastrol as a promising candidate for the prevention and control of PED and other swine viral infections.

## 1. Introduction

Porcine epidemic diarrhea (PED) is a highly contagious, acute intestinal disease that affects pigs of all ages, with particularly devastating impacts on suckling piglets. Caused by the PED virus (PEDV), the disease is characterized by severe diarrhea, vomiting, and dehydration, with mortality rates in suckling piglets approaching 100% in severe outbreaks [1]. First identified in the United Kingdom in 1971, PEDV belongs to the *Alphacoronavirus* genus in the Coronaviridae family [2, 3]. It has a linear, single‐stranded, positive‐sense RNA genome that is organized in the following order: 5’UTR, replicase polyprotein 1a/b (ORF1a/b), spike (S), ORF3, envelope (E), membrane (M), nucleocapsid (N), and 3’UTR [4]. A key challenge in PED vaccine development is that immune pressure from vaccination can induce mutations in the S gene, enabling the virus to evade host immunity [5]. Recent studies have demonstrated that commercial vaccines fail to provide adequate protection against epidemic strains due to such genetic mutations [6, 7, 8, 9]. These issues underscore the urgent need to explore antiviral agents as an alternative strategy for effective and comprehensive disease control.

In recent years, compounds originating from plants have garnered escalating attention for their therapeutic potential, particularly in combating viral infections like PEDV. Notably, numerous contemporary medications have been developed from bioactive plant compounds [10]. The search for plant‐derived antiviral agents dates back to the 1950s, when scientists assessed more than 200 plant species for their efficacy against influenza A virus in embryonated eggs [11]. More recently, compounds including quercetin [12–14], bis‐benzylisoquinoline alkaloids [15–17], glycyrrhizin [18, 19], griffithsin [20], and levistolide A [21], have demonstrated anti‐PEDV activity. Despite these advances, no commercial anti‐PEDV drugs available, highlighting the need for further exploration of natural products as antiviral candidates.

Celastrol, also known as tripterine, is a natural pentacyclic triterpenoid extracted from the traditional Chinese medicinal plant *Tripterygium wilfordii*. Extensive pharmacological studies have revealed that celastrol exerts a wide range of therapeutic activities, including immunomodulatory, anti‐inflammatory, antitumor, anti‐fibrotic, and antiviral effects [22–24], along with protective benefits for the cardiovascular, cerebrovascular, and nervous systems [25–28]. Celastrol demonstrates antiviral activity against a variety of viruses through multiple mechanisms. For example, celastrol reduces equine alphaherpesvirus Type 8 replication by activating the Nrf2/HO‐1 pathway [29], inhibits dengue virus replication by inducing IFN‐*α* expression and activating JAK‐STAT signaling [30], and suppresses influenza A virus [31], hepatitis C virus [32], human immunodeficiency virus [33], and human parvovirus [34]. Notably, celastrol shows strong antiviral activity against SARS‐CoV‐2, a betacoronavirus, by binding to the viral S protein, inhibiting 3CL (Pro) activity, reducing viral replication, decreasing IL‐6 secretion, and alleviating pulmonary lesions in severe COVID‐19 patients [35–38]. These findings suggest that celastrol is a promising antiviral agent with potential applicability against a range of viruses, including the *Alphacoronavirus* PEDV. In this study, we explored its antiviral‐PEDV activities and elucidated the underlying mechanism.

In this study, we explored the anti‐PEDV activities of celastrol in Vero E6 cells. By conducting network pharmacology analysis on the hub proteins between celastrol and virus, we focused on the apoptosis pathway and revealed that celastrol‐induced reactive oxygen species (ROS)mediated apoptosis is a crucial mechanism underlying its anti‐PEDV effect. Moreover, we confirmed the antiviral activity of celastrol against two other significant porcine viruses, porcine deltacoronavirus (PDCoV) and porcine reproductive and respiratory syndrome virus (PRRSV), in vitro. These findings suggest that celastrol holds considerable promise as a therapeutic agent for PEDV infection within the pig farming industry.

## 2. Materials and Methods

### 2.1. Cell Culture and Virus

African green monkey kidney epithelial cells (Vero E6; ATCC CRL‐1586), porcine kidney‐15 cells (PK‐15; ATCC CCL‐33), and monkey embryonic kidney epithelial cells (Marc‐145; ATCC CRL‐12231) cells were cultured in Dulbecco’s modified eagle medium (DMEM; GIBCO), supplemented with 10% fetal bovine serum (FBS; EvaCell). The PEDV strain CV777 (GenBank: KT323979.1) was grown and titrated in Vero E6 cells, the PDCoV strain HKU15 (GenBank: NC_039208.1) was grown and titrated in PK‐15 cells, and PRRSV strain BJ4 (GenBank: AF331831.1) was grown and titrated in Marc‐145 cells.

### 2.2. Antibodies and Reagents

Mouse monoclonal antibodies of PEDV N, PDCoV N, and PRRSV N were stored from our laboratory. GAPDH mouse monoclonal antibody (Cat#AF0006), *α*‐tubulin mouse monoclonal antibody (Cat#AF2827), *β*‐actin mouse monoclonal antibody (Cat#AF0003), HRP‐conjugated goat anti‐mouse IgG (Cat#A0216), and HRP‐conjugated goat anti‐rabbit IgG (Cat#A0208) were purchased from Beyotime Biotechnology. Bcl‐2 (124) mouse monoclonal antibody (Cat#15071), Caspase‐3 (D3R6Y) rabbit monoclonal (Cat#14220) antibody, and cleaved Caspase‐3 (Asp175) rabbit monoclonal (Cat#9664) were purchased from Cell Signaling Technology. The goat anti‐mouse Alexa Fluor 488 conjugate antibody (Cat#HS231‐01) was purchased from TransGen Biotech. The goat anti‐rabbit Alexa Fluor 594 (Cat#A‐11012) was purchased from Thermo Fisher Scientific.

Celastrol (Cat#T3028) and apoptosis inducer staurosporine (Cat#T6680) were purchased from TargetMol. Caspase inhibitor Z‐VAD‐FMK (Cat#C1202) was purchased from Beyotime Biotechnology.

### 2.3. Cytotoxicity Assay

The cytotoxicity of celastrol was evaluated by using an MTT assay. Briefly, Vero E6, PK‐15, and Marc‐145 plated in 96‐well plates were treated with increasing concentrations (from 0.25 to 2 μM) of celastrol (TargetMol, Cat#T3028) for 24 h at 37°C in 5% CO_2_. Then the culture medium was removed and replaced with 100 μL MTT (3‐[4,5‐dimethyl‐2‐thiazolyl]‐2,5‐diphenyl tetrazolium bromide, 0.5 mg/mL) and incubated at 37°C for 4 h. After removal of the supernatant, 150 μL DMSO were added in each wells to dissolve the formazan crystals for 10 min. Optical density (OD) was measured at 490 nm using a microplate reader. GraphPad Prism 8.0 (GraphPad Software, San Diego, CA) was used to calculate half‐maximal cytotoxic concentrations (CC_50_).

### 2.4. Indirect Immunofluorescence Assay (IFA)

Cells were washed with PBS, followed by fixation with 4% paraformaldehyde (PFA) for 20 min. Subsequently, 0.25% (V/V) Triton X‐100 was added to each well for 30 min at room temperature (RT) to permeabilize the cell membrane. After three PBS rinses, cells underwent blocking with PBS containing 5% bovine serum albumin (BSA) at RT for 1 h. The PEDV N mouse monoclonal antibody diluted 1/500 was incubated at 4°C overnight, and goat anti‐mouse Alexa Fluor 488 conjugate antibody diluted 1/1000 was applied at RT for 1 h. The cell nucleus was stained with 4’,6‐diamidino‐2‐phenylindole (DAPI). Image acquisition was performed using a Olympus CKX53 fluorescence microscope.

### 2.5. Western Blotting Assay

Cells were lysed in RIPA lysis buffer containing 1 mM phenylmethylsulfonylfluoride (PMSF) at 4°C. The supernatant was harvested by centrifugation at 10,000 *g* for 20 min at 4°C. Equal amounts of cell lysates were loaded onto sodium dodecyl sulfate–polyacrylamide (SDS‐PAGE) gels and then transferred to a PVDF membrane. The membranes were blocked by incubation with 5% skim milk powder for 1 h at RT. Then PEDV N mouse monoclonal antibody diluted 1/500 was incubated at 4°C overnight and HRP‐conjugated goatanti‐mouse IgG diluted 1/1000 for 1 h at RT.

### 2.6. Reverse Transcription‐Quantitative Polymerase Chain Reaction (RT‐qPCR)

RNA was extracted from samples using a total RNA rapid extraction kit (Fastagen, Shanghai, China) and reverse transcripted as complementary DNA (cDNA) using a PrimeScript RT reagent kit with gDNA Eraser (Takara Bio). Gene expression in cDNA samples was measured by ChamQ Universal SYBR qPCR Master Mix according to the manufacturer’s instruction (Vazyme Biotech). The sequences of primers used for RT‐qPCR are listed in Table 1. Quantitative real‐time PCR was performed on the CFX96 real‐time PCR detection system (Bio‐Rad). The RNA relative expression of each target gene was normalized to GAPDH expression and then calculated using the 2‐^*△△*CT^ method.

**Table 1:** Sequences of primers used for RT‐qPCR.

Primers name	Sequence (5′‐3′)
PEDV‐N‐F	CCGTGGTGAGCGAATTGAAC
PEDV‐N‐R	TCAGACGCCTTTCTGACACC
PDCoV‐S‐F	ATATGGCTTGCCATTGCCCT
PDCoV‐S‐R	AAAGGACGGTGTTGGTTGGT
PRRSV‐ORF7‐F	AGATCATCGCCCAACAAAAC
PRRSV‐ORF7‐R	GACACAATTGCCGCTCACTA
Monkey‐GAPDH‐F	CCTTCCGTGTCCCTACTGCCAAC
Monkey‐GAPDH‐R	GACGCCTGCTTCACCACCTTCT
Porcine‐GAPDH‐F	GAAGGTCGGAGTGAACGGATTT
Porcine‐GAPDH‐R	TGGGTGGAATCATACTGGAACA

### 2.7. TCID _50_ Assay

The titers of all viral samples in this study were determined using the TCID_50_ method. Specifically, Vero E6 ells were seeded in 96‐well plates and incubated at 37°C. After discarding the supernatant, cells were infected with 10‐fold serially diluted samples (test group) or DMEM medium (blank control group) for 4 days prior to observation of the presence of cytopathic effect. Both the test and blank control groups comprised eight replicate wells each, and TCID_50_ was calculated employing the Reed and Muench method.

### 2.8. Antiviral Activity Assay

The antiviral activity assay was performed to evaluate in vitro PEDV‐inhibiting capacities. Vero E6 cell monolayers grown in 24‐well plates were preincubated by celastrol with different concentrations for 4 h and infected with PEDV (multiplicity of infection [MOI] = 0.1) for 1 h at 37°C. Supernatants were removed and fresh medium containing different concentrations of celastrol was then added. Cells and supernatants were then collected at 24 h postinfection (hpi), and the antiviral activity was determined by RT‐qPCR assay, western blotting assay, TCID_50_ assay and IFA, respectively.

### 2.9. Sustained Antiviral Activity Assay

Vero E6 cell monolayers grown in 24‐well plates were preincubated by celastrol with different concentrations for 4 h and infected with PEDV with MOI = 0.1 for 1 h at 37°C. Supernatants were removed and fresh medium containing different concentrations of celastrol was then added. Cells and supernatants were then collected at 12, 24, and 36 hpi, and the antiviral activity was determined by RT‐qPCR assay, western blotting assay, and TCID_50_ assay respectively.

### 2.10. Anti‐PDCoV and PRRSV Assay

For the experiment on the inhibitory activity of celastrol against PDCoV, PK‐15 cell monolayers grown in 24‐well plates were preincubated by celastrol with different concentrations for 4 h and infected with PDCoV with MOI = 0.1 for 1 h at 37°C. Supernatants were removed and fresh medium containing different concentrations of celastrol was then added. Cells and supernatants were then collected at 24 hpi, and the antiviral activity was determined by RT‐qPCR assay and western blotting assay.

For the experiment on the inhibitory activity of celastrol against PRRSV, Marc‐145 cell monolayers grown in 24‐well plates were preincubated by celastrol with different concentrations for 4 h and infected with PRRSV with MOI = 0.1 for 1 h at 37°C. Supernatants were removed and fresh medium containing different concentrations of celastrol was then added. Cells and supernatants were then collected at 24 hpi, and the antiviral activity was determined by RT‐qPCR assay and western blotting assay.

### 2.11. Time‐of‐Addition Assay

The 10^5^ Vero E6 cells were seeded in 24‐well plates and then infected with the PEDV at an MOI of 0.1 for 1 h at 37°C. Drugs were pretreated (preincubation), cotreated (during‐time), post treated (post time), or treated all over the time (full‐time) during the PEDV infection. For the preincubation group, cells were incubated with 0.5 μM of celastrol for 4 h at 37°C, followed by three time washes with PBS, and then infected with PEDV (MOI = 0.1) for 1 h at 37°C. For the during‐time group, cells were simultaneously incubated with PEDV and 0.5 μM of celastrol for 1 h at 37°C. After that, the virus–drug mixture was removed, and the cells were washed three times with PBS prior to the complete medium being added. For the post‐time group, cells were first infected with PEDV for 1 h at 37°C. After that, the virus was removed, and the cells were washed three times with PBS prior to the complete medium containing 0.5 μM of celastrol being added. For the full‐time group, cells were initially incubated with a 0.5 μM concentration of celastrol for 4 h. Subsequently, the solution was discarded, and the cells were washed three times with PBS. Then, the cells were concurrently exposed to PEDV and 0.5 μM of celastrol for 1 h at 37°C. Following this, the virus was removed, and the cells were washed three times with PBS before being treated with complete medium containing 0.5 μM of celastrol. At 24 hpi, samples were collected to determine virus titer by an endpoint dilution assay and PEDV N gene mRNA level by RT‐qPCR.

### 2.12. Virucidal Assay

PEDV (1 MOI) and 1 μM celastrol or isopyknic DMSO were mixed for 1 h at 37°C. To eliminate the potential effects of the celastrol on PEDV infection, the mixture was diluted 100‐fold and immediately added to Vero E6 cells. After 72 h, the virus was collected and titred using TCID_50_ assay.

### 2.13. Network Pharmacology

Three hundred seventy‐nine targets of celastrol were obtained from the TCMSP database (https://old.tcmsp-e.com/tcmsp.php), SwissTargetPrediction database (http://www.swisstargetprediction.ch/), and CTD database (https://ctdbase.org/). Additionally, using “virus” as the keyword and after filtering with a “Relevance” score greater than two, 3830 disease‐related targets were retrieved from the GeneCards database. A venn diagram of the intersection targets between celastrol and viruses was constructed using the WeiShengXin website (https://www.bioinformatics.com.cn/), identifying 238 overlapping targets. The 238 intersecting targets of celastrol and viruses were then imported into the DAVID database (https://davidbioinformatics.nih.gov/) for GO and KEGG enrichment analyses to obtain biological processes (BP), cellular components (CC), molecular functions (MF), and signaling pathway information related to the antiviral effects of celastrol. Bar charts for the top‐ranked GO enrichment analyses and bubble charts for KEGG pathway enrichment were also generated using the WeiShengXin website. Furthermore, the 238 intersecting targets were imported into the STRING database (https://cn.string-db.org/), with the interaction score set at 0.4. The protein–protein interaction (PPI) network was visualized using Cytoscape 3.10.3, and the degree values of each gene in the PPI network were calculated.

### 2.14. Molecular Docking Method

The protein crystal structures of MAPK1 (PDB ID: 4QTA), MAPK3 (PDB ID: 3FHR), IL6 (PDB ID: 1ALU), CASP3 (PDB ID: 1GFW), AKT1 (PDB ID: 4QTA), IL1B (PDB ID: 1HIB), MAPK8 (PDB ID: 2XRW), NFKB1 (PDB ID: 1LE5), TP53 (PDB ID: 4MZR), and MAPK14 (PDB ID: 2FST) were all downloaded from the PDB database (https://www.rcsb.org/). The molecular structure of celastrol (PubChem CID: 122724) was downloaded from the PubChem database (https://pubchem.ncbi.nlm.nih.gov/). The protein structures were prepared for docking by removing any excess organic ligands and water molecules using PyMOL software. Docking grids for each target site were generated using AutoDock. By comparing the binding modes of the original ligands, semiflexible docking parameters were set to identify the complex conformations with the most favorable binding free energies for celastrol. The docking results were visualized in three dimensions using PyMOL software, providing a clear representation of the interactions between celastrol and its target proteins.

### 2.15. Determination of the Levels of Intracellular ROS

ROS was determined using the DCFH‐DA ROS Assay Kit (Solarbio, Beijing, China). Briefly, Vero E6 plated in 12‐well plates were infected by PEDV (MOI = 0.1) for 1 h at 37°C in 5% CO_2_, after discarding the supernatant, cells were treated with or without celastrol for 24 h. Vero E6 cells were rinsed thrice using PBS and stained using 10 µM DCFH‐DA (dissolved in DMEM) for 45 min. Rosup (50 mg/mL) was uesd as positive control. ROS was detected at the FITC channel. The positive‐staining cells were analyzed using fluorescence microscopy.

### 2.16. Statistical Analysis

All data were analyzed using GraphPad Prism 8.0 software (GraphPad Software, San Diego, CA) and are presented as the mean ± standard error of the mean of at least three independent experiments. Statistical comparisons between groups were performed using Student’s *t*‐tests when comparing the means of two independent or paired groups, whereas two‐way ANOVA was used to analyze the effects of two categorical independent variables and their interaction on a continuous dependent variable. Two‐tailed *p*‐values were determined.

## 3. Results

### 3.1. Celastrol Inhibits PEDV Infection in a Dose‐Dependent Manner

The chemical structures of celastrol are shown in Figure 1A. Cytotoxicity testing in Vero E6 cells using MTT assay revealed a half‐maximal cytotoxic concentration (CC_50_) of 1.583 μM (Figure 1B). The antiviral effect of celastrol against PEDV was examined by western blotting, RT‐qPCR assay, TCID_50_, and IFA. As shown in Figure 1C,D, celastrol significantly inhibited PEDV N protein expression and suppressed PEDV N mRNA levels in a dose‐dependent manner, with significant inhibition observed at concentrations of 0.1–0.5 μM at 24 hpi. Consistently, celastrol significantly reduced PEDV titers, determined by cytopathic effect‐based endpoint dilution assay (Figure 1E). IFA further confirmed a dose‐dependent reduction in the proportion of virus‐infected cells (Figure 1F). These results indicate that celastrol effectively inhibits PEDV replication in Vero E6 cells while exhibiting minimal cytotoxicity.

Figure 1Cytoxicity and anti‐PEDV activity of celastrol in Vero E6 cells. (A) Chemical structures of celastrol. (B) Cytoxicity of celastrol in Vero E6 cells at 24 hpi using MTT assay, and CC_50_ values were calculated using GraphPad Prism 8.0. (C) Celastrol inhibits PEDV N expression in Vero E6 cells. (D) Celastrol suppresses PEDV N gene transcription. (E) Celastrol reduces PEDV titers, as determined by TCID_50_. (F) Antiviral activity of celastrol against PEDV is determined by IFA. scale bar: 100 μm. Each datum represents the mean ± SD of three independent experiments (*n* = 3). Statistical significance was determined using Student’s *t*‐tests.

**Figure:**
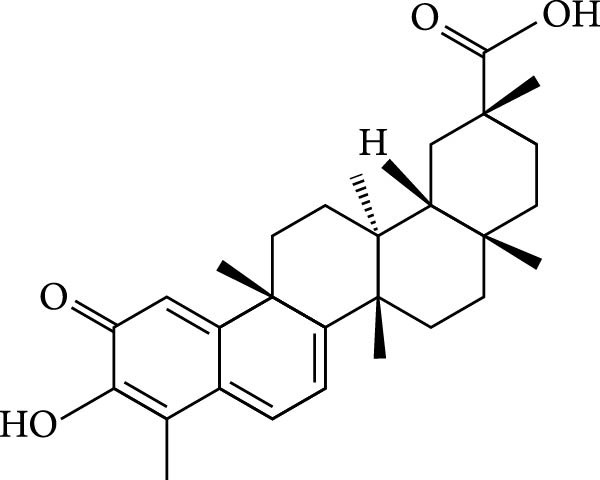
(A)

**Figure:**
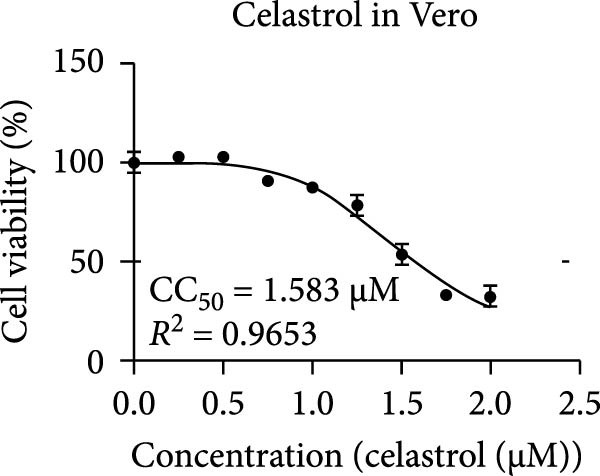
(B)

**Figure:**
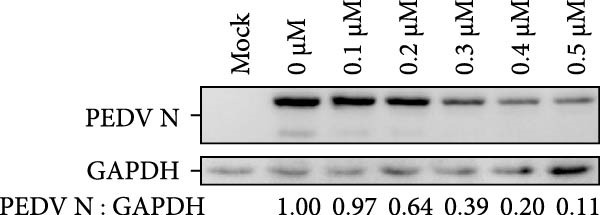
(C)

**Figure:**
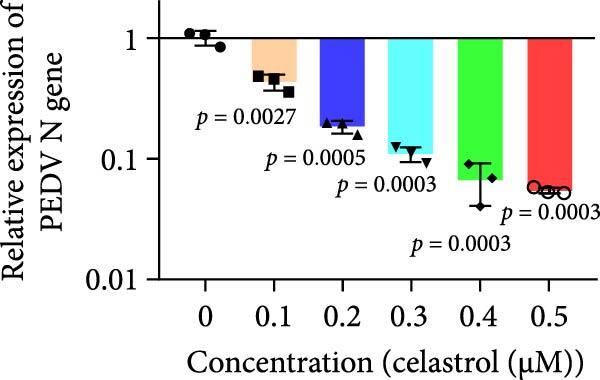
(D)

**Figure:**
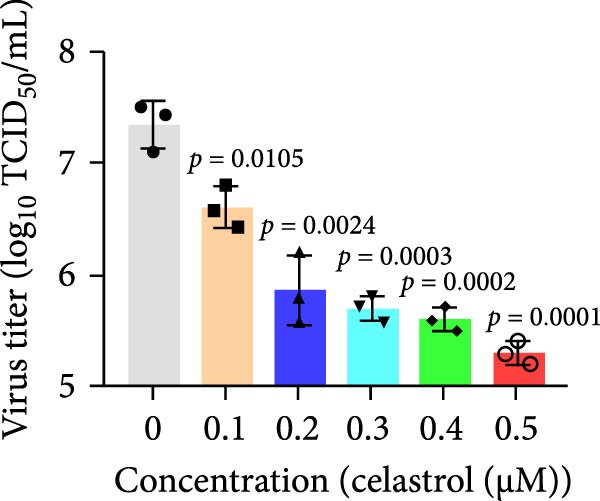
(E)

**Figure:**
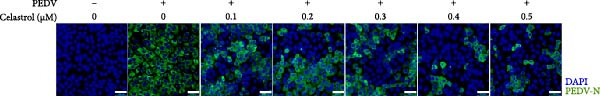
(F)

### 3.2. Celastrol Exhibits Sustained Antiviral Activity Against PEDV

The antiviral effects of celastrol were further determined at 12, 24, and 36 hpi. RT‐qPCR analysis showed that the mRNA levels of PEDV N gene were significantly inhibited by celastrol at all examined time points (Figure 2A). Likewise, TCID_50_ assays showed reduced progeny PEDV titers following celastrol treatment (Figure 2B). Western blotting analysis demonstrated a consistent reduction in PEDV N protein expression at all three time points in the presence of celastrol (Figure 2C), and densitometric analysis further validated this downregulation (Figure 2D). Taken together, these data suggest that celastrol exhibits a sustained inhibitory effect on PEDV replication and protein synthesis throughout the infection cycle.

Figure 2Antiviral effects of celastrol at different times postinfection against PEDV in Vero E6 cells. (A) Celastrol inhibits PEDV N gene transcription at 12, 24, and 36 hpi in Vero E6 cells as determined by RT‐qPCR. (B) Celastrol inhibits PEDV progeny virus production at the indicated time points, measured by TCID_50_ assay. (C) Celastrol reduces PEDV N protein expression, detected by western blotting. (D) Densitometric analysis of PEDV N levels using ImageJ software; values were normalized to the virus control at 12 hpi. Each datum represents the results of three independent experiments (mean ± SD, *n* = 3). The data was then analyzed using a two‐way ANOVA with GraphPad Prism 8.0.

**Figure:**
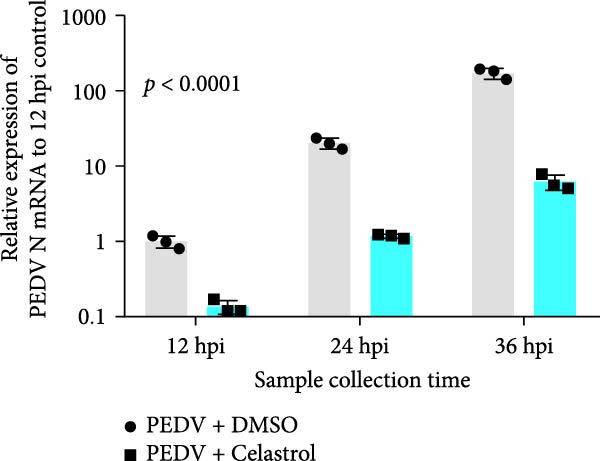
(A)

**Figure:**
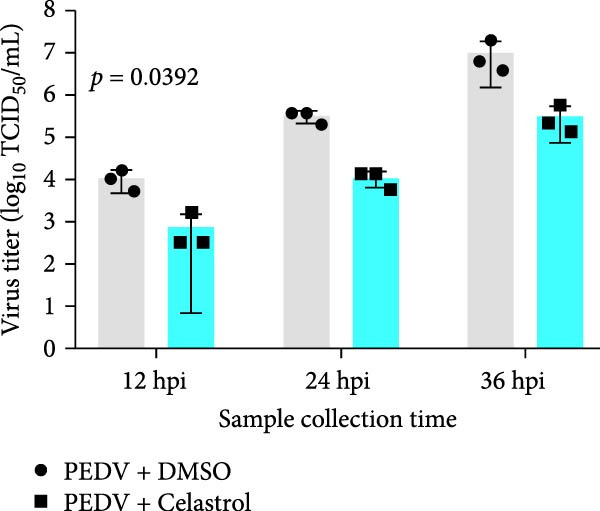
(B)

**Figure:**
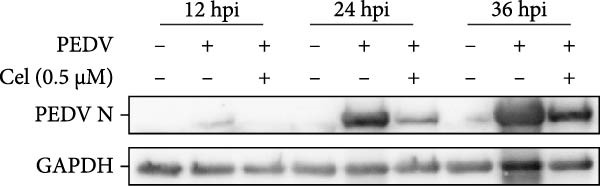
(C)

**Figure:**
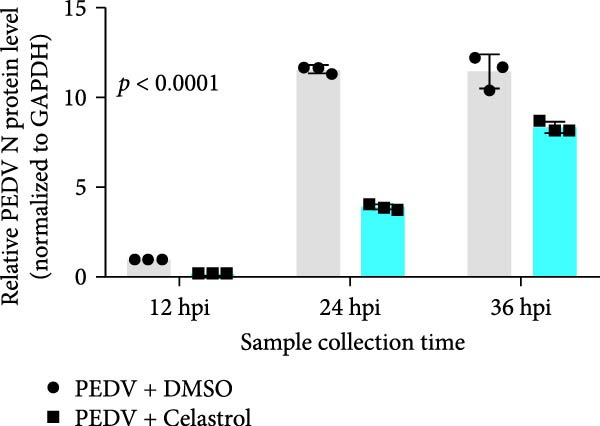
(D)

### 3.3. Celastrol Suppresses PEDV Infection Across Different Infection Doses

The antiviral effects of celastrol were evaluated at MOI of 0.01, 0.1, and 1. RT‐qPCR analysis showed that celastrol significantly inhibited the mRNA levels of PEDV N gene at all MOIs (Figure 3A). Consistently, TCID_50_ assay revealed that celastrol reduced the PEDV progeny virus titers (Figure 3B), and western blotting analysis confirmed decreased N protein expression (Figure 3C). Densitometric analysis further supported the downregulation (Figure 3D). Taken together, these results suggest that celastrol effectively inhibits PEDV replication and protein synthesis across a range of viral inoculation doses.

Figure 3Antiviral effects of celastrol against PEDV at different infection doses in Vero E6 cells. (A) Celastrol inhibits PEDV N gene transcription at MOIs of 0.01, 0.1, and 1, measured by RT‐qPCR. (B) Celastrol inhibits PEDV progeny virus production at the indicated MOIs, determined by TCID_50_ assay. (C) Celastrol reduces PEDV N protein expression, detected by western blotting. (D) Densitometric analysis of N protein levels using ImageJ software, normalized to the 0.01 MOI infection control. Each datum represents the results of three independent experiments (mean ± SD, *n* = 3). The data was then analyzed using a two‐way ANOVA with GraphPad Prism 8.0.

**Figure:**
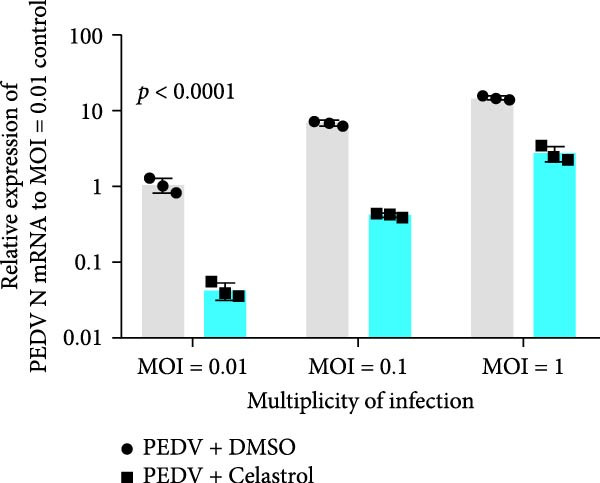
(A)

**Figure:**
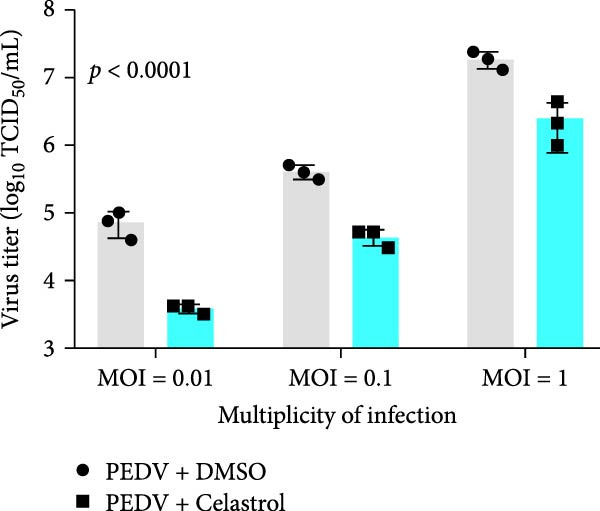
(B)

**Figure:**
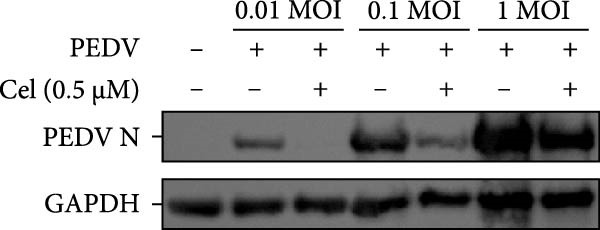
(C)

**Figure:**
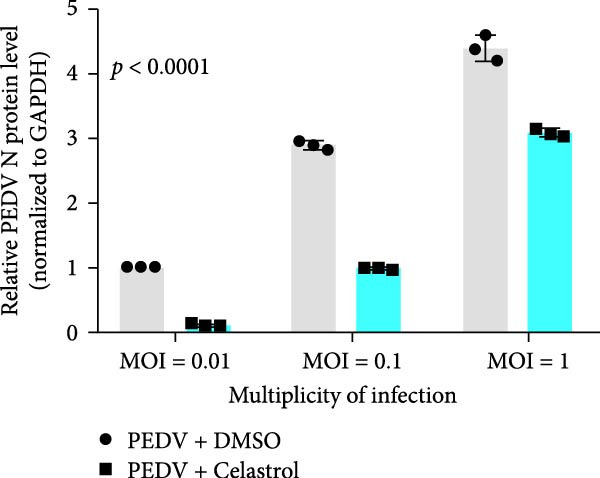
(D)

### 3.4. Celastrol Targets the Post entry Stage of PEDV Infection

We conducted the time‐of‐addition assay to explore which stage of the PEDV life cycle is affected by celastrol (Figure 4A). Vero E6 cells were treated with 0.5 μM celastrol in five groups: control (infection only), full time (−4–24 hpi), preincubation (−4–0 hpi), during‐entry (0–1 hpi), and post entry (1–24 hpi). RT‐qPCR analysis showed dramatical decrease in PEDV N gene transcription in the full‐time and post‐entry groups, but not in the preincubation or during‐entry groups (Figure 4B). Similarly, TCID_50_ assays revealed significantly lower progeny virus titers in the full‐time and post‐entry groups compared with controls (Figure 4C). As shown in Figure 4D, western blotting analysis confirmed decreased PEDV N protein expression under the same conditions. Consistently, IFA showed markedly reduced viral fluorescence signals (Figure 4E). In addition, a virucidal assay further demonstrated that celastrol did not directly inactivate PEDV (Figure 4F). To clarify the specific post entry stage targeted by celastrol, we separately detected viral particles in the intracellular and extracellular compartments, finding that celastrol does not inhibit PEDV assembly or release (Figure 4G–I). We also conducted IFA staining for PEDV dsRNA, and the results indicated that celastrol significantly reduced the fluorescence intensity and density of PEDV dsRNA (Figure 4J). These results indicate celastrol primarily inhibits PEDV replication at the post entry stage of infection.

Figure 4Celastrol inhibits PEDV infection at post entry stage in Vero E6 cells. (A) Schematic diagram of the time‐of‐addition assay. (B) PEDV N gene transcription levels in different groups. measured by RT‐qPCR. (C) Progeny virus titers in different groups, determined by TCID_50_ assay. (D) PEDV N expression in the different groups, evaluated by western blotting. (E) Antiviral stage of celastrol against PEDV, determined by IFA (scale bar: 50 μm). (F) virucidal activity of celastrol against PEDV was determined by a TCID_50_ assay. Progeny virus titers in extracellular (G) and intracellular (H) compartments, determined by TCID_50_ assay. (I) The ratio of extracellular to intracellular virus titer was calculated to assess virion release efficiency. (J) Vero E6 cells infected with PEDV at an MOI of 0.1 were treated with celastrol and subsequently fixed and stained with a dsRNA antibody at 24 hpi (scale bar: 50 μm). Each datum represents the results of three independent experiments (mean ± SD, *n* = 3). The data were compared using Student’s *t*‐tests.

**Figure:**
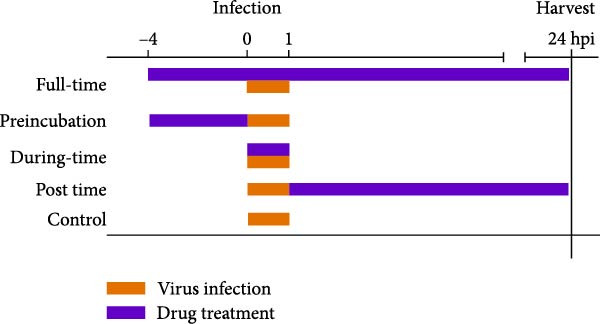
(A)

**Figure:**
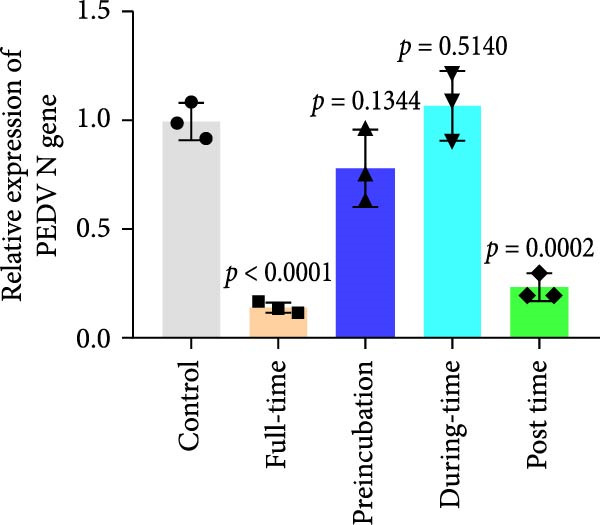
(B)

**Figure:**
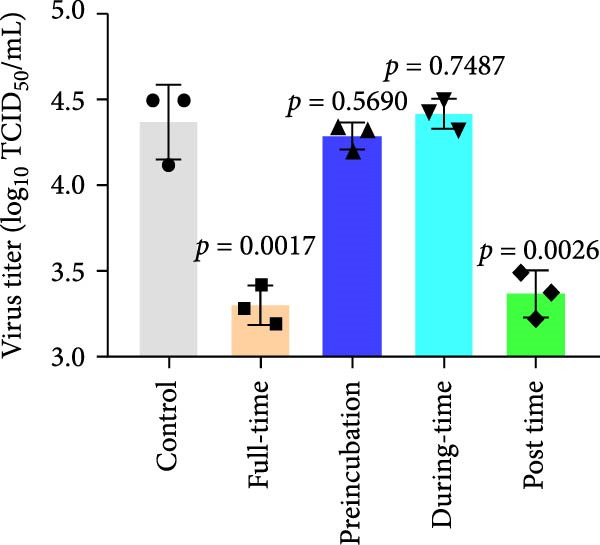
(C)

**Figure:**
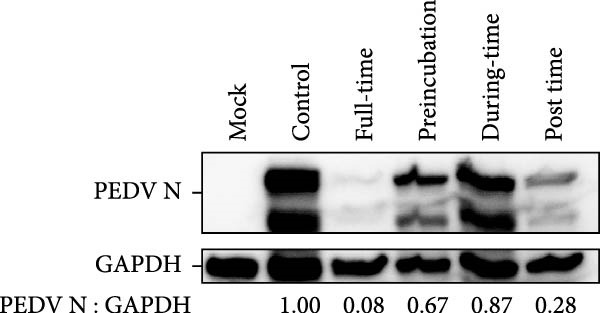
(D)

**Figure:**
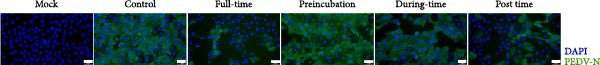
(E)

**Figure:**
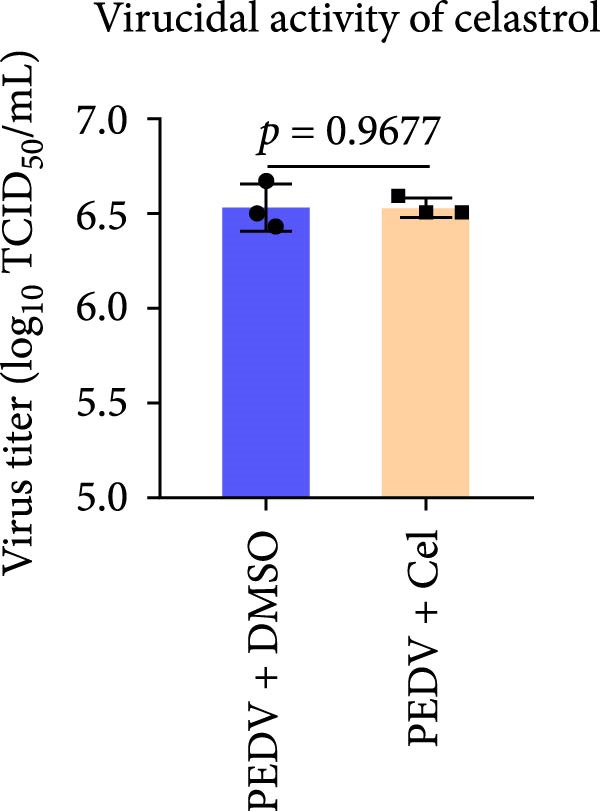
(F)

**Figure:**
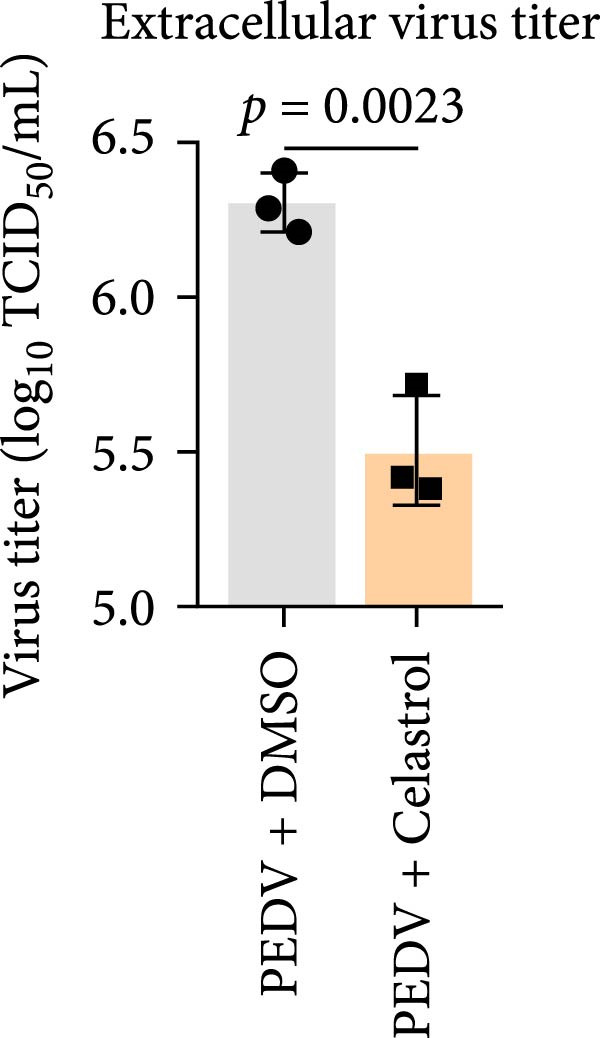
(G)

**Figure:**
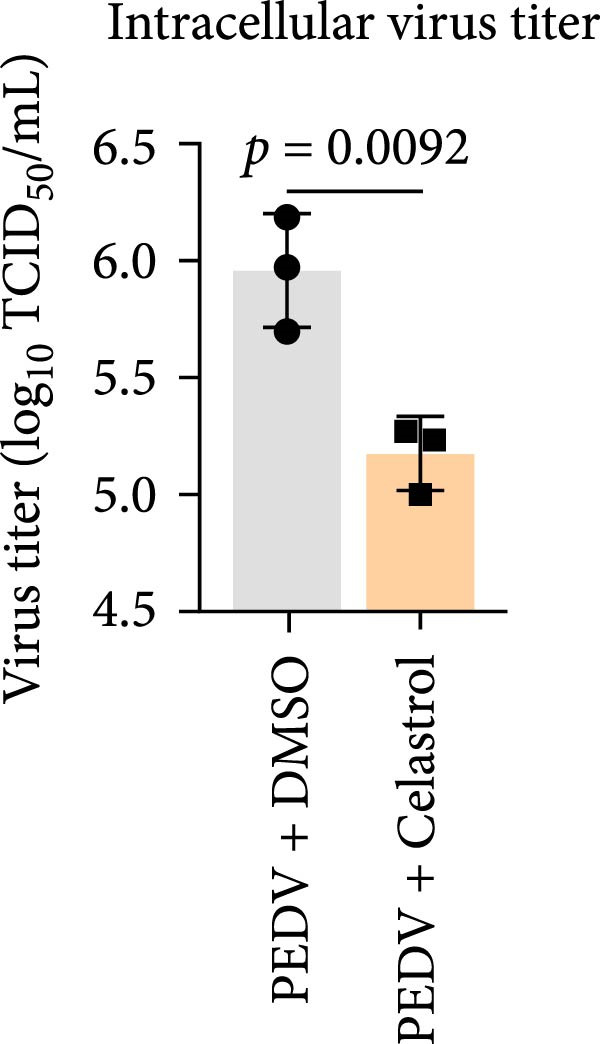
(H)

**Figure:**
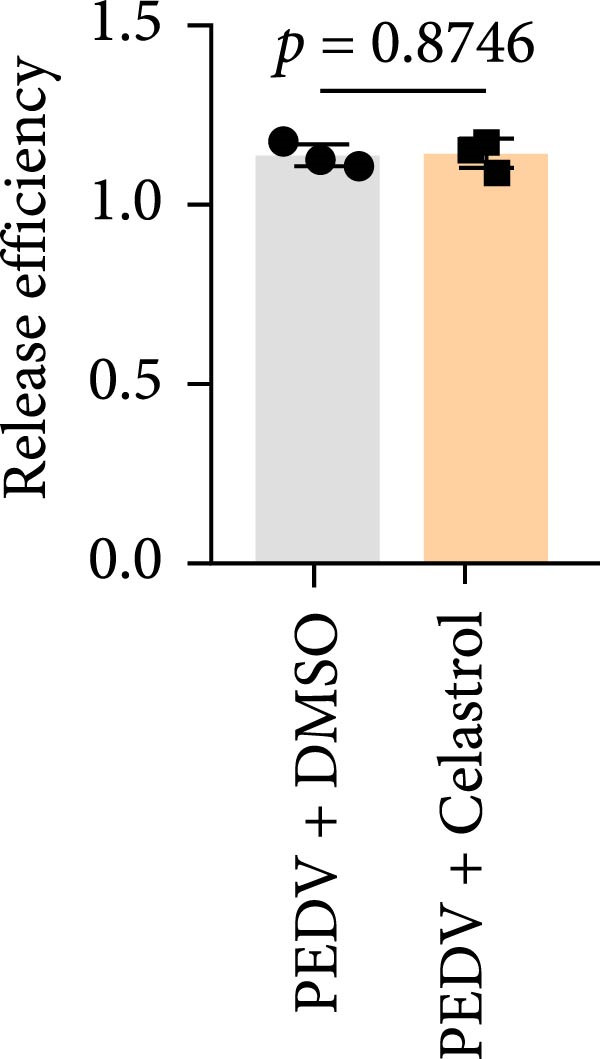
(I)

**Figure:**
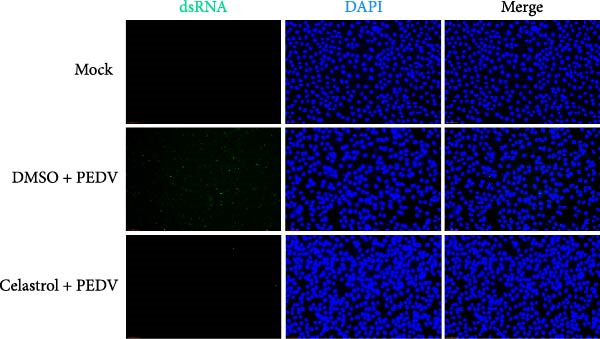
(J)

### 3.5. Network Pharmacology Implicates Apoptosis Signaling in Celastrol Activity

Target prediction via the SwissTargetPrediction and SymMap databases identified 379 potential celastrol targets. Using the Genecards, CTD, and TTD databases, 3830 virus‐related targets were obtained. Intersection analysis revealed 238 overlapping targets potentially involved in celastrol’s antiviral effects (Figure 5A). GO and KEGG enrichment analyses were performed on these common targets, and a PPI network with 183 nodes was constructed using Cytoscape 3.10.3. The top 10 hub proteins ranked by degree included MAPK1, MAP3, IL6, CASP3, AKT1, IL1B, MAPK8, NFKB1, and TP53 (Figure 5B). The binding affinity of celastrol for the top 10 hub proteins was evaluated via AutoDock docking analysis, with the binding energies presented in Table 2. Additionally, the docking models illustrated that celastrol forms hydrogen bonds with these proteins (Supporting Information: Figure S1).

Figure 5Network pharmacology analysis of celastrol targets against PEDV. (A) Venndiagram showing the overlap between celastrol targets and virus‐related targets. (B) PPI network of common targets constructed using Cytoscape 3.9.0. (C) GO analysis of common targets. (D) The KEGG analysis of common targets.

**Figure:**
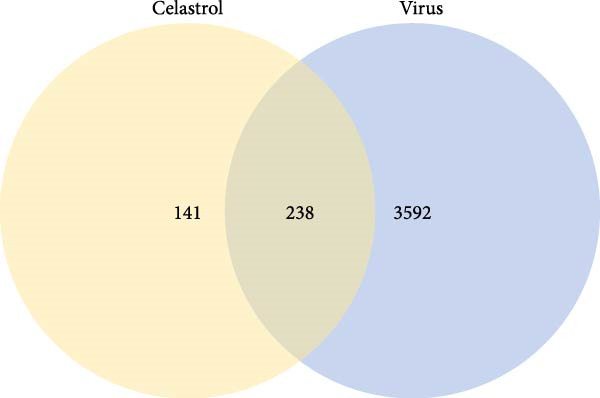
(A)

**Figure:**
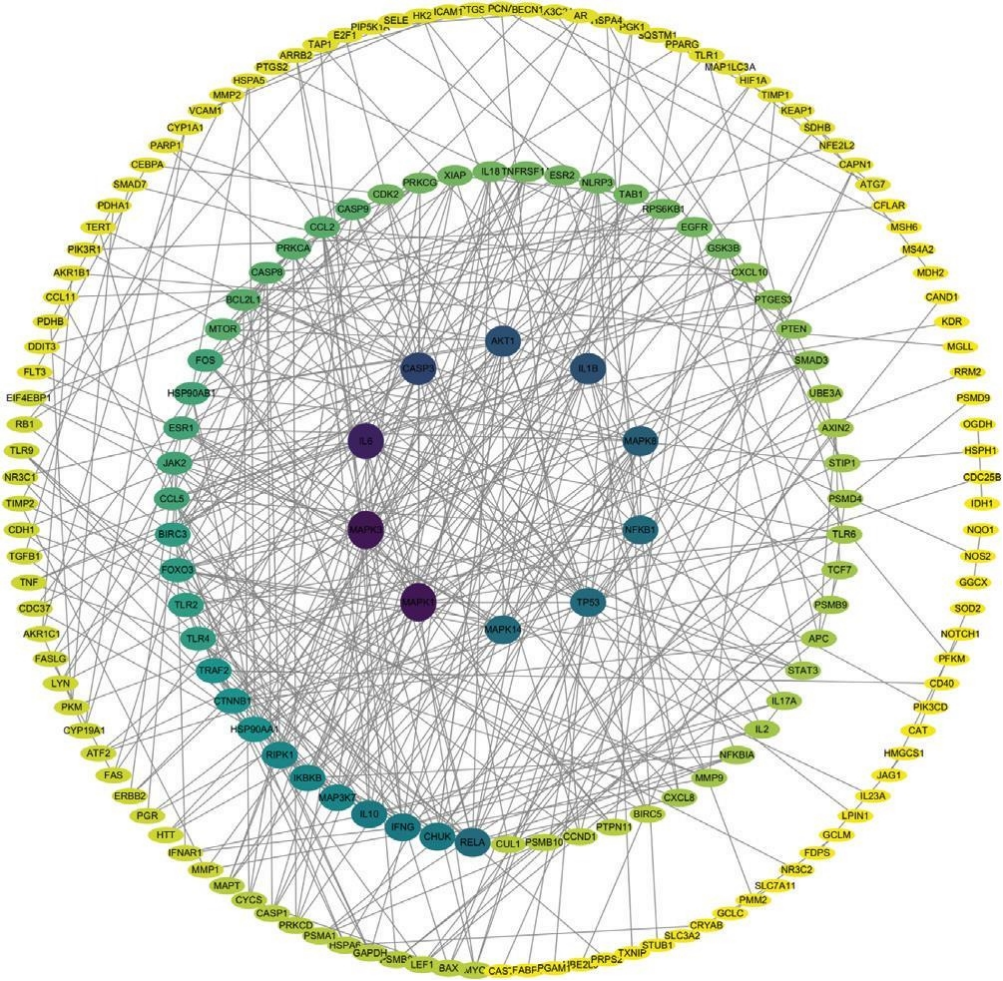
(B)

**Figure:**
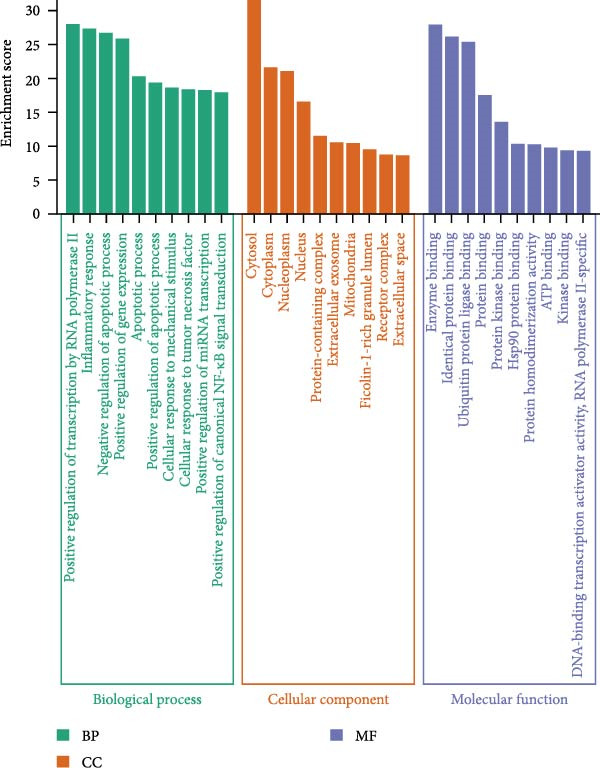
(C)

**Figure:**
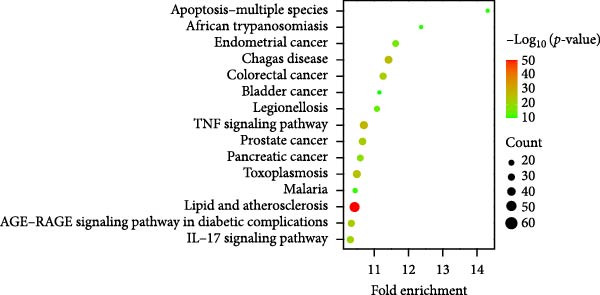
(D)

**Table 2:** Binding free energy of celastrol with the top 10 hub proteins.

Active component	Binding free energy/(kJ/mol)									
	IL6	CASP3	AKT1	MAPK1	IL1B	MAPK3	MAPK8	MAPK14	TP53	NFKB1
Celastrol	−6.8	−8.2	−8.0	−7.9	−8.1	−8.0	−7.6	−8.0	−8.9	−8.4

GO enrichment suggested that the inhibition of PEDV replication by celastrol may be associated with processes such as positive regulation of transcription by RNA polymerase II, inflammatory response, regulation of apoptosis, gene expression, cellular response to mechanical stimulus and tumor necrosis factor, miRNA transcription, and NF‐*κ*B signal transduction (Figure 5C). KEGG enrichment analysis further highlighted apoptosis as the most significantly enriched pathway (Figure 5D).

### 3.6. Celastrol Induces ROS‐Mediated Apoptosis to Inhibit PEDV

Network pharmacology analysis identified the apoptosis signaling pathway as the primary target of celastrol, prompting further investigation into its role in anti‐PEDV activity. Western blotting analysis exhibited reduced pro‐caspase‐3 levels in PEDV‐infected cells at 24 hpi relative to mock‐infected controls, indicating caspase‐3 cleavage (Figure 6A,B). Celastrol treatment further enhanced this cleavage in a dose‐dependent manner, yielding a prominent cleaved‐caspase‐3 fragment. Notably, celastrol alone induced caspase‐3 activation in a dose‐dependent manner, comparable to the apoptosis inducer staurosporine, and this effect was abolished by the pan‐caspase inhibitor Z‐VAD‐FMK (Figure 6C), confirming that celastrol can intrinsically trigger apoptosis. Moreover, Bcl‐2 expression declined in all PEDV‐infected groups, regardless of celastrol treatment (Figure 6D). Collectively, these data support that celastrol enhances PEDV‐induced apoptosis.

Figure 6Celastrol inhibits PEDV infection through promoting PEDV‐triggered apoptosis. (A) Western blotting analysis of PEDV N, caspase‐3, cleaved caspase‐3, and Bcl‐2 proteins in PEDV‐infected Vero E6 cells treated with celastrol, apoptosis inducer staurosporine or pan‐caspase inhibitor Z‐VAD‐FMK at 24 hpi. (B–D) Quantification of the band intensities for caspase‐3, cleaved caspase‐3, and Bcl‐2 proteins to *β*‐actin was measured by imageJ software and data were normalized by comparing to the mock group. (E) Intracellular ROS levels were detected by DCF fluorescence intensity, scale bar = 50 μM. (F) PEDV N gene transcription level in Vero E6 cells under celastrol and NAC treatment was detected by RT‐qPCR. (G) PEDV titer in Vero E6 cells under celastrol and NAC treatment was detected by TCID_50_ assay. (H) PEDV N protein level in Vero E6 cells under celastrol and NAC treatment was detected by western blotting assay. Each datum represents the results of three independent experiments (mean ± SD, *n* = 3). The data were compared using Student’s *t*‐tests.

**Figure:**
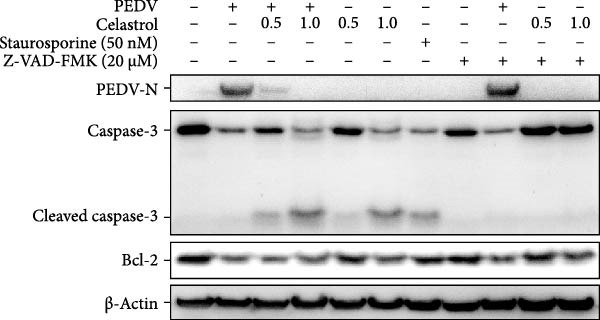
(A)

**Figure:**
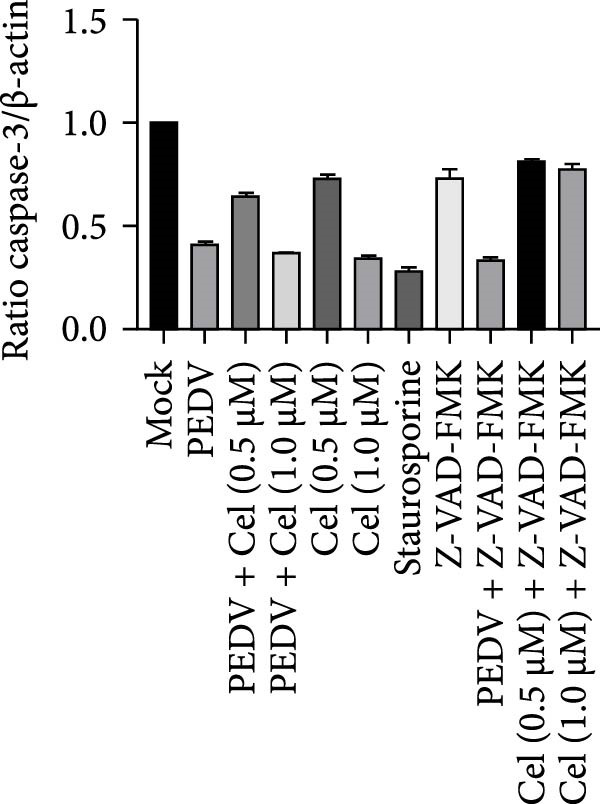
(B)

**Figure:**
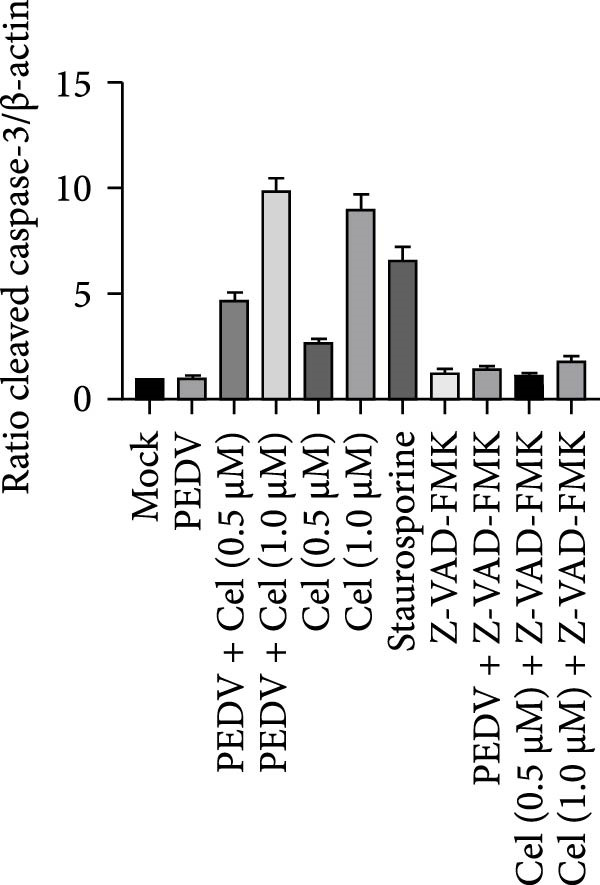
(C)

**Figure:**
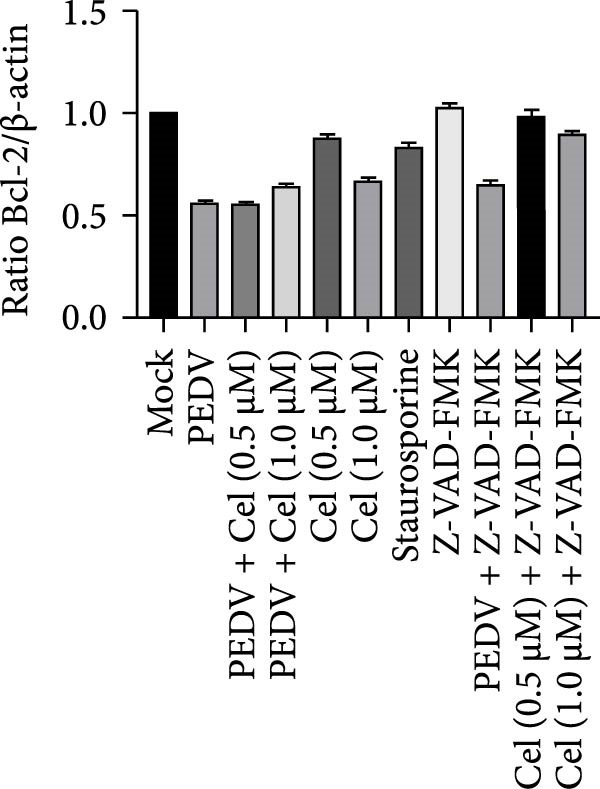
(D)

**Figure:**
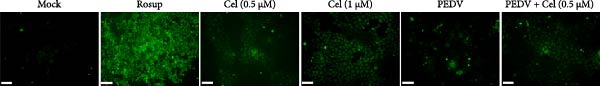
(E)

**Figure:**
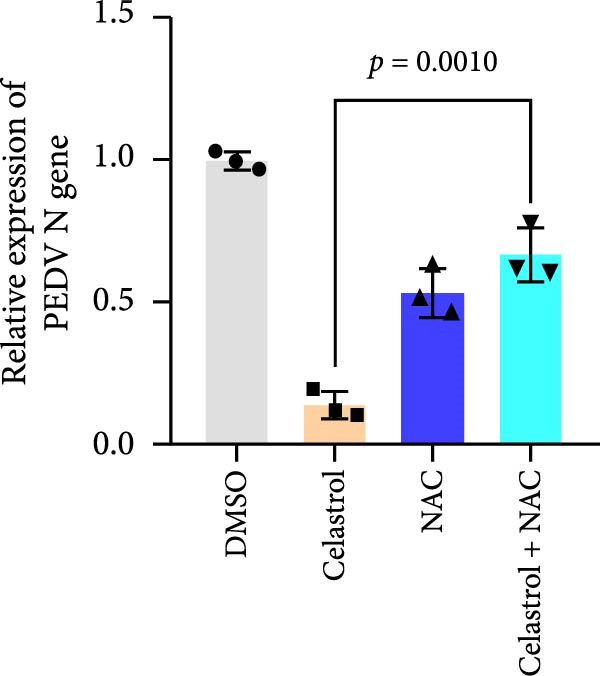
(F)

**Figure:**
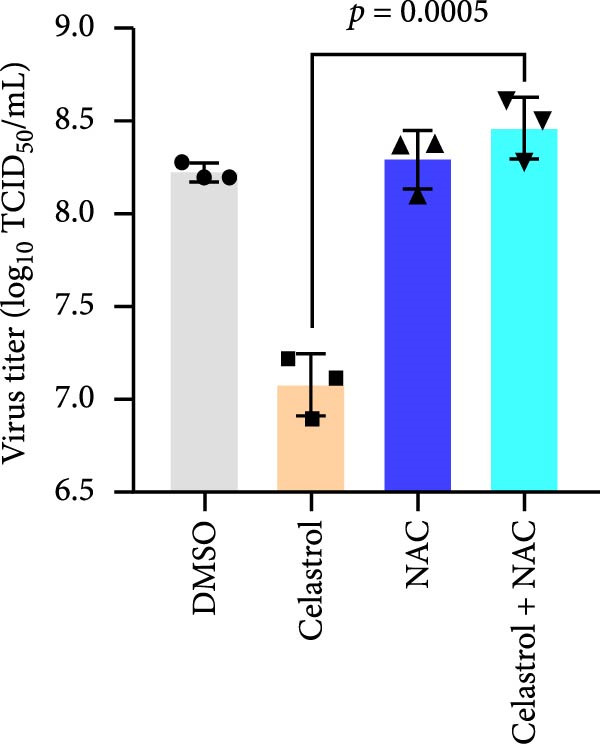
(G)

**Figure:**
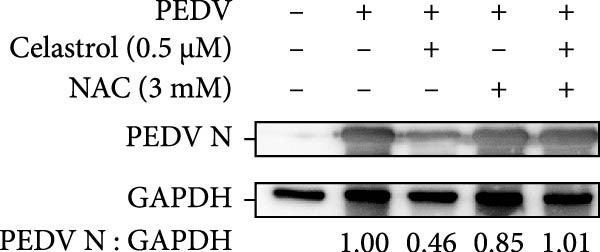
(H)

Given that excessive accumulation of intracellular ROS can trigger apoptosis, we next investigate the role of ROS in the context of celastrol treatment [39]. Compared with the mock group, celastrol dose‐dependently induced ROS accumulation and further amplified PEDV‐induced ROS production, with Rosup serving as a positive control (Figure 6E). To further explore the relationship between ROS accumulation and the antiviral activity of celastrol, we coincubated cells with celastrol and the ROS inhibitor N‐acetylcysteine (NAC). The results of RT‐qPCR, TCID_50_, and western blotting assay showed that NAC abolished celastrol’s inhibitory effects on viral RNA synthesis, viral titer, and N protein expression (Figure 6F–H). In addition, we detected the levels of ROS and apoptosis markers in PEDV‐infected cells at 12 and 24 hpi (Supporting Information: Figure S2A). The results showed that ROS levels rise at 24 hpi in PEDV‐infected cells. Treatment with celastrol enhances ROS levels starting at 12 hpi and continuing through 24 hpi, with no significant increase afterwards. Moreover, at 24 hpi, celastrol‐treated PEDV‐infected cells exhibit greater caspase‐3 activation compared to 12 hpi, suggesting that ROS production precedes caspase‐3 activation (Supporting Information: Figure S2B).

We further investigated whether the apoptosis inhibitor Z‐VAD‐FMK could, like NAC, reverse the antiviral activity of celastrol. We conducted cotreatment experiments with Z‐VAD‐FMK and celastrol. MTT assays showed that treatment with Z‐VAD‐FMK alone or cotreatment with Z‐VAD‐FMK and celastrol did not cause significant cytotoxicity (Supporting Information: Figure S3A). However, coincubation of celastrol with the apoptosis inhibitor Z‐VAD‐FMK did not eliminate its antiviral activity against PEDV, as evidenced by unchanged levels of PEDV N protein (Supporting Information: Figure S3B) and viral RNA (Supporting Information: Figure S3C), indicating that celastrol’s antiviral effect is primarily mediated through ROS regulation rather than direct induction of apoptosis.

These findings suggest that celastrol inhibits PEDV replication by inducing ROS accumulation, which in turn promotes apoptosis.

### 3.7. Celastrol Shows Antiviral Activity Against PDCoV and PRRSV

Clinical PEDV infections are frequently accompanied by coinfections with other viruses [40]. To evaluate the broader antiviral potential of celastrol, we tested its activity against PDCoV, another coronavirus, and PRRSV, a major swine pathogen. As the in vitro replication of PDCoV and PRRSV is typically evaluated in PK‐15 and Marc‐145 cells, respectively [41, 42], we first assessed the cytotoxicity of celastrol in these cell lines. The CC_50_ of celastrol in PK‐15 cells was 1.695 μM (Figure 7A) and 2.638 µM in Marc‐145 cells (Figure 7D), indicating lower toxicity in the latter. Celastrol significantly inhibited PDCoV S mRNA and protein expression in PK‐15 cells in a dose‐dependent manner (0.1–0.5 μM, Figure 7B,C). Similarly, as shown in Figure 7E,F, celastrol exhibited a dose‐dependent inhibition of PRRSV ORF7 mRNA and N protein expression in Marc‐145 cells within the same concentration range. These results indicate that celastrol exerts the antiviral effect against both PDCoV and PRRSV.

Figure 7Celastrol’s antiviral activity against PDCoV and PRRSV and cytotoxicity in PK‐15 and Marc‐145 Cells. (A) Cytoxicity of celastrol in PK‐15 cells, and CC_50_ was calculated using GraphPad Prism 8.0. (B) Celastrol inhibited PDCoV S gene transcription in PK‐15 cells. (C) Celastrol inhibited PEDV N expression in PK‐15 cells. (D) Cytoxicity of celastrol was examined in Marc‐145 cells. (E) Celastrol inhibited PRRSV ORF7 gene transcription in Marc‐145 cells. (F) celastrol inhibited PRRSV N expression in Marc‐145 cells. Each datum represents the results of three independent experiments (mean ± SD, *n* = 3). The data were compared using Student’s *t*‐tests.

**Figure:**
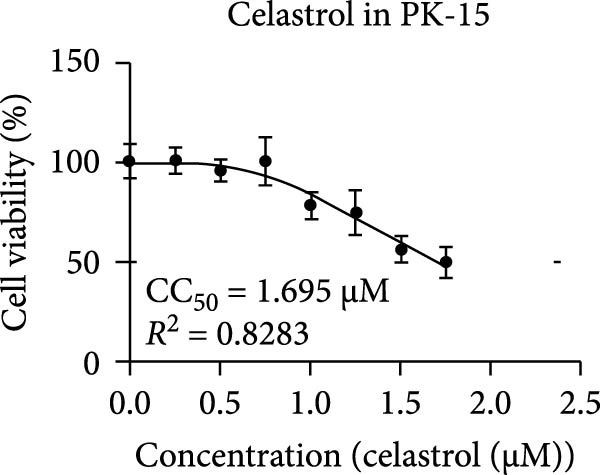
(A)

**Figure:**
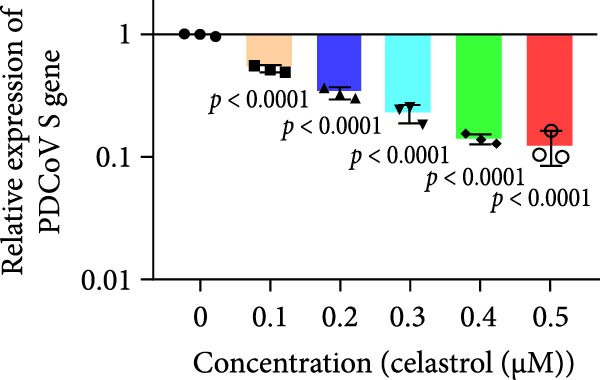
(B)

**Figure:**
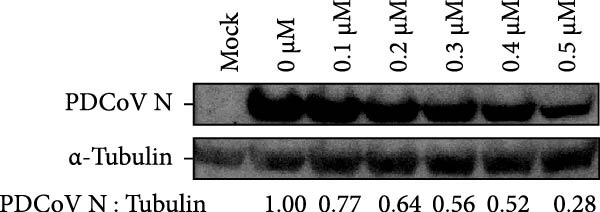
(C)

**Figure:**
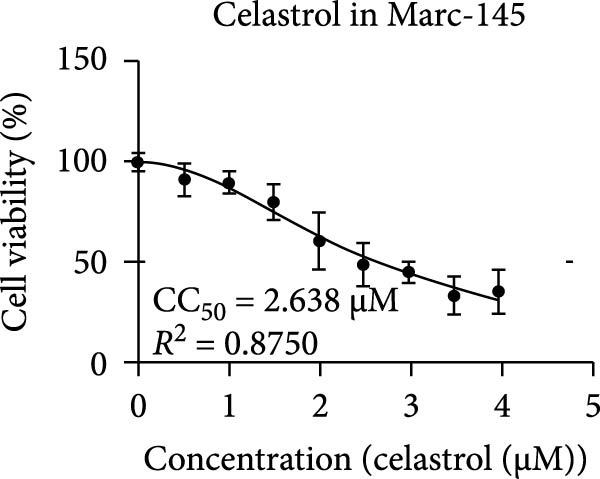
(D)

**Figure:**
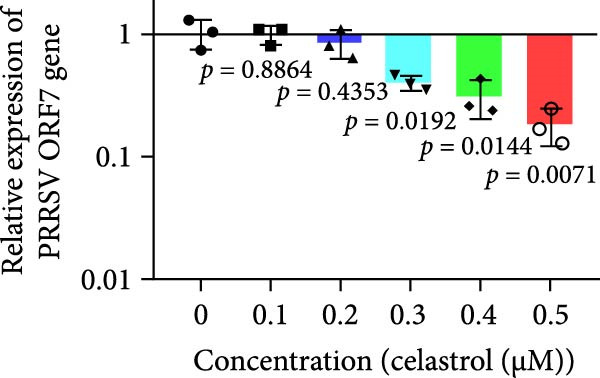
(E)

**Figure:**
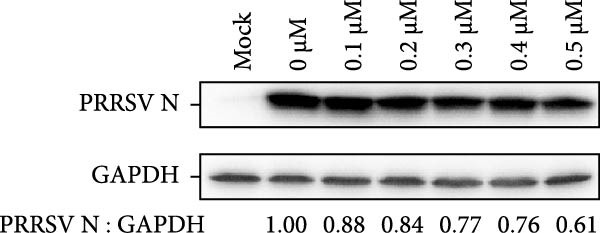
(F)

## 4. Discussion

PEDV infection and its ability to evade the immune system pose significant challenges to vaccine development. Therefore, it is crucial to explore alternative strategies, such as antiviral agents, for prevention and control of PEDV. Recent advances in antiviral drug development against PEDV have shown considerable promise, exploring the mechanisms of these agents and identifying novel targets to control measures.

Here, we demonstrated the antiviral activity of celastrol, which inhibits PEDV replication and promotes ROS‐mediated apoptosis at the post entry stage. Earlier research has shown that celastrol exhibits virucidal effects on SARS‐CoV‐2 by directly binding S protein [35]. However, our findings indicated that celastrol inhibits the post entry stages of PEDV but no inhibitory effects on its entry or direct inactivating effects. Given that PEDV and SARS‐CoV‐2 are both coronaviruses, these findings suggest that the antiviral mechanisms of celastrol likely differ between these viruses.

Apoptosis constitutes an intrinsic host defense mechanism that curtails viral replication by eliminating infected cells. However, PEDV bidirectionally regulates apoptosis to modulate apoptosis for its own benefit: it suppresses apoptosis in infected cells to create a conducive environment for progeny virus replication, while simultaneously exploiting apoptosis to expedite the release and dissemination of progeny virions [43]. Meanwhile, PEDV induces apoptosis via the AIF pathway and DR5 receptor‐activated Caspase‐8 pathway, and inhibiting AIF and DR5 expression significantly disrupts PEDV‐induced apoptosis and viral replication [44]. Conversely, PEDV encodes several viral proteins that play crucial roles in inhibiting apoptosis. For example, the PEDV ORF3 protein inhibits apoptosis in infected cells to promote its proliferation within host cells [45]. PEDV nsp9 interacts with host histone 2 cluster H2BE to inhibit ER stress‐mediated apoptosis induced by PEDV, thereby promoting viral replication [46]. Thus, the relationship between PEDV infection and apoptosis remains controversial. Our findings demonstrate that PEDV induces apoptosis, an effect that is significantly enhanced by celastrol. Similar observations have been reported for other PEDV inhibitors. For example, triacetyl resveratrol has been shown to inhibit PEDV by inducing early apoptosis, thereby eliminating PEDV‐infected cells [47].

During apoptosis, mitochondrial and other organelle dysfunction boosts ROS production, which in turn amplifies apoptosis via a positive feedback loop [48]. According to previous studies, celastrol could trigger ROS‐mediated apoptosis and increasing the levels of ROS [49]. Simultaneously, PEDV strain activates the PERK/eIF2*α* pathway in Vero E6 cells, leading to enhanced ROS generation [50]. Consistent with our findings, a similar antiviral mechanism was observed with levistolide A, which inhibits PEDV replication by inducing ROS generation and stimulating endoplasmic reticulum stress [21]. However, coincubation of celastrol with the apoptosis inhibitor Z‐FAM‐VAD did not eliminate its antiviral activity against PEDV, suggesting that celastrol’s antiviral effect primarily involves ROS regulation rather than direct apoptosis induction. For example, studies have shown that ROS generated during respiratory syncytial virus infection can activate the host’s antiviral immune response, while inhibiting ROS production weakens this response [51]. This suggests that ROS may play a more critical role in celastrol’s antiviral mechanism, with apoptosis being merely a downstream effect of ROS regulation.

However, there are still unresolved issues in this study that require further in‐depth research in the future. For example, we failed to identify the drug targets of celastrol. In‐depth exploration of celastrol’s antiviral targets against PEDV, along with a comprehensive grasp of its mechanisms, not only supports its clinical application but also clarifies the interplay between PEDV and its host. Recent advancements in proteomics, including chemical proteomics, protein microarray, degradation‐based protein profiling, proteome‐wide label‐free approaches, network pharmacology, target‐based drug screening, multiomics analysis, and hypothesis‐driven target confirmation, have shed light on the multifaceted mechanisms of action of celastrol [52]. The identification of numerous targets for celastrol underscores its potential as a multitarget therapeutic agent. Subsequent analyses, including network interactions and frequency assessments of these targets, point to PRDX [53], HMGB1 [54], HSP90 [55], STAT3 [56], and PKM2 [57] as likely key targets for celastrol. Whether these reported targets of celastrol are related to its anti‐PEDV capability, or whether they play a significant role in PEDV infection, requires further in‐depth investigation in the future. On the other hand, evaluating the in vivo efficacy of antiviral drugs is crucial. However, we have not yet conducted in vivo experiments to assess the antiviral efficacy of celastrol against PEDV. Celastrol has been used in clinical research, and current reports indicate minimal adverse reactions and no significant safety concerns [58, 59]. In summary, future research on celastrol’s anti‐PEDV properties should prioritize identifying drug targets, clarifying specific anti‐PEDV mechanisms, and assessing antiviral effects in pigs.

In addition, we also evaluated the antiviral effects of celastrol against PRRSV and PDCoV in vitro, which suggests that celastrol may hold promise as an antiviral agent against a broader range of swine viruses. However, due to the lack of experimental materials, other important swine viruses, such as African swine fever virus, classical swine fever virus, and swine influenza virus, have not been further verified. Further validation of the antiviral effects of celastrol against more porcine viruses in the future will provide a more comprehensive theoretical basis for the commercialization of celastrol as an antiviral drug.

In conclusion, our study has demonstrated the antiviral activity of celastrol, revealed its inhibitory effects on PEDV replication, and for the first time, shown the involvement of ROS in its antiviral mechanism. These findings hold significant implications for the prevention and control of PED. Our research provides valuable insights for identifying potential drug targets, which could aid in the development of new interventions.

## 5. Conclusions

This study demonstrated that celastrol inhibits PEDV replication by promoting ROS‐mediated apoptosis, as revealed through comprehensive in vitro experiments and network pharmacology analysis. The findings highlight celastrol’s potential as an antiviral agent effective against PEDV, offering a novel theoretical basis for the development of anti‐PEDV drugs and contributing to the comprehensive control of PEDV infections.

## Conflicts of Interest

The authors declare no conflicts of interest.

## Author Contributions

Junhai Zhu and Kaifang Yang contributed equally to this work.

## Funding

This study was supported by Grants from the Natural Science Foundation of Chongqing (Grant CSTB2024NSCQ‐MSX1131), the Natural Science Foundation of Chongqing (Grant CSTB2024NSCQ‐MSX0467), the Fundamental Research Funds for the Central Universities (Grant SWU‐KQ24043), and the National Center of Technology Innovation for Pigs (Grant NCTIP‐XD/B11).

## Supporting Information

Additional supporting information can be found online in the Supporting Information section.

## Supporting information


**Supporting Information** Figure S1: Binding conformations of celastrol bound to the top 10 hub proteins generated by virtual ligand docking. Figure S2: Time‐dependent effects of celastrol on ROS and apoptosis in PEDV‐Infected Cells. Figure S3: Co‐incubation with Z‐FAM‐VAD failed to impact celastrol’s antiviral efficacy against PEDV.

## Data Availability

The data that support the findings of this study are available from the corresponding author upon reasonable request.

## References

[1] Jung K. , Saif L. J. , and Wang Q. , Porcine Epidemic Diarrhea Virus (PEDV): An Update on Etiology, Transmission, Pathogenesis, and Prevention and Control, Virus Research. (2020) 286, 10.1016/j.virusres.2020.198045, 198045. 32502552 PMC7266596

[2] Jung K. , Annamalai T. , Lu Z. , and Saif L. J. , Comparative Pathogenesis of US Porcine Epidemic Diarrhea Virus (PEDV) Strain PC21A in Conventional 9-Day-Old Nursing Piglets vs. 26-Day-Old Weaned Pigs, Veterinary Microbiology. (2015) 178, no. 1-2, 31–40, 10.1016/j.vetmic.2015.04.022, 2-s2.0-84930475593. 25939885 PMC7117181

[3] Rosas-Murrieta N. H. , Rodríguez-Enríquez A. , Herrera-Camacho I. , Millán-Pérez-Peña L. , Santos-López G. , and Rivera-Benítez J. F. , Comparative Review of the State of the Art in Research on the Porcine Epidemic Diarrhea Virus and SARS-CoV-2, Scope of Knowledge Between Coronaviruses, Viruses. (2024) 16, no. 2, 10.3390/v16020238, 238. 38400014 PMC10892376

[4] Lin F. , Zhang H. , and Li L. , et al.PEDV: Insights and Advances Into Types, Function, Structure, and Receptor Recognition, Viruses. (2022) 14, no. 8, 10.3390/v14081744, 1744. 36016366 PMC9416423

[5] Van Diep N. , Choijookhuu N. , and Fuke N. , et al.New Tropisms of Porcine Epidemic Diarrhoea Virus (PEDV) in Pigs Naturally Coinfected by Variants Bearing Large Deletions in the Spike (S) Protein and PEDVs Possessing an Intact S Protein, Transboundary and Emerging Diseases. (2020) 67, no. 6, 2589–2601, 10.1111/tbed.13607. 32356614

[6] Guo W. , Wang C. , and Song X. , et al.Immunogenicity and Protective Efficacy of a Trimeric Full-Length S Protein Subunit Vaccine for Porcine Epidemic Diarrhea Virus, Vaccine. (2024) 42, no. 4, 828–839, 10.1016/j.vaccine.2024.01.020. 38220489

[7] Li Z. , Ma Z. , Li Y. , Gao S. , and Xiao S. , Porcine Epidemic Diarrhea Virus: Molecular Mechanisms of Attenuation and Vaccines, Microbial Pathogenesis. (2020) 149, 10.1016/j.micpath.2020.104553, 104553. 33011361 PMC7527827

[8] Schumacher L. , Chen Q. , and Fredericks L. , et al.Evaluation of the Efficacy of an S-INDEL PEDV Strain Administered to Pregnant Gilts Against a Virulent Non-S-INDEL PEDV Challenge in Newborn Piglets, Viruses. (2022) 14, no. 8, 10.3390/v14081801, 1801. 36016423 PMC9416680

[9] Yao X. , Qiao W.-T. , and Zhang Y.-Q. , et al.A New PEDV Strain CH/HLJJS/2022 Can Challenge Current Detection Methods and Vaccines, Virology Journal. (2023) 20, no. 1, 1–13, 10.1186/s12985-023-01961-z. 36670408 PMC9859669

[10] Zhu J. , Huang L. , and Gao F. , et al.Berbamine Hydrochloride Inhibits African Swine Fever Virus Infection In Vitro, Molecules. (2023) 28, no. 1, 10.3390/molecules28010170, 170. PMC9822360 36615369

[11] Chantrill B. H. , Coulthard C. E. , Dickinson L. , Inkley G. W. , Morris W. , and Pyle A. H. , The Action of Plant Extracts on a Bacteriophage of Pseudomonas Pyocyanea and on Influenza A Virus, Journal of General Microbiology. (1952) 6, no. 1-2, 74–84, 10.1099/00221287-6-1-2-74, 2-s2.0-0006321811. 14927853

[12] Choi H.-J. , Kim J.-H. , and Lee C.-H. , et al.Antiviral Activity of Quercetin 7-Rhamnoside Against Porcine Epidemic Diarrhea Virus, Antiviral Research. (2009) 81, no. 1, 77–81, 10.1016/j.antiviral.2008.10.002, 2-s2.0-57149102069. 18992773 PMC7114206

[13] Gong T. , Wu D. , and Feng Y. , et al.Inhibitory Effects of Quercetin on Porcine Epidemic Diarrhea Virus In Vitro and In Vivo, Virology. (2024) 589, 10.1016/j.virol.2023.109923, 109923. 37977082

[14] Song J. H. , Shim J. K. , and Choi H. J. , Quercetin 7-Rhamnoside Reduces Porcine Epidemic Diarrhea Virus Replication via Independent Pathway of Viral Induced Reactive Oxygen Species, Virology Journal. (2011) 8, no. 1, 10.1186/1743-422X-8-460, 2-s2.0-80053448061. PMC3200163 21967756

[15] Dong S. , Yu R. , and Wang X. , et al.Bis-Benzylisoquinoline Alkaloids Inhibit Porcine Epidemic Diarrhea Virus In Vitro and In Vivo, Viruses. (2022) 14, no. 6, 10.3390/v14061231, 1231. 35746702 PMC9228057

[16] Zhang C. , Chen H. , and Sun L. , et al.Bis-Benzylisoquinoline Alkaloids Inhibit Porcine Epidemic Diarrhea Virus by Disrupting Virus Entry, Pathogens. (2023) 12, no. 6, 10.3390/pathogens12060845, 845. 37375535 PMC10304817

[17] Zhang W. , Shen H. , and Wang M. , et al.Fangchinoline Inhibits the PEDV Replication in Intestinal Epithelial Cells via Autophagic Flux Suppression, Frontiers in Microbiology. (2023) 14, 10.3389/fmicb.2023.1164851, 1164851. 37485535 PMC10360400

[18] Gao R. , Zhang Y. , and Kang Y. , et al.Glycyrrhizin Inhibits PEDV Infection and Proinflammatory Cytokine Secretion via the HMGB1/TLR4-MAPK p38 Pathway, International Journal of Molecular Sciences. (2020) 21, no. 8, 10.3390/ijms21082961, 2961. 32340172 PMC7215578

[19] Huan C.-C. , Wang H.-X. , Sheng X.-X. , Wang R. , Wang X. , and Mao X. , Glycyrrhizin Inhibits Porcine Epidemic Diarrhea Virus Infection and Attenuates the Proinflammatory Responses by Inhibition of High Mobility Group Box-1 Protein, Archives of Virology. (2017) 162, no. 6, 1467–1476, 10.1007/s00705-017-3259-7, 2-s2.0-85011844413. 28175983 PMC7086885

[20] Li L. , Yu X. , and Zhang H. , et al.In Vitro Antiviral Activity of Griffithsin Against Porcine Epidemic Diarrhea Virus, Virus Genes. (2019) 55, no. 2, 174–181, 10.1007/s11262-019-01633-7, 2-s2.0-85060141112. 30637608 PMC7089098

[21] Zeng W. , Ren J. , and Li Z. , et al.Levistolide A Inhibits PEDV Replication via Inducing ROS Generation, Viruses. (2022) 14, no. 2, 10.3390/v14020258, 258. 35215851 PMC8878026

[22] Astry B. , Venkatesha S. H. , and Laurence A. , et al.Celastrol, a Chinese Herbal Compound, Controls Autoimmune Inflammation by Altering the Balance of Pathogenic and Regulatory T Cells in the Target Organ, Clinical Immunology. (2015) 157, no. 2, 228–238, 10.1016/j.clim.2015.01.011, 2-s2.0-84924544171. 25660987 PMC4410084

[23] Luo P. , Liu D. , and Zhang Q. , et al.Celastrol Induces Ferroptosis in Activated HSCs to Ameliorate Hepatic Fibrosis via Targeting Peroxiredoxins and HO-1, Acta Pharmaceutica Sinica B. (2022) 12, no. 5, 2300–2314, 10.1016/j.apsb.2021.12.007. 35646542 PMC9136576

[24] Zhu Y. , Liu X. , Zhao P. , Zhao H. , Gao W. , and Wang L. , Celastrol Suppresses Glioma Vasculogenic Mimicry Formation and Angiogenesis by Blocking the PI3K/Akt/mTOR Signaling Pathway, Frontiers in Pharmacology. (2020) 11, 10.3389/fphar.2020.00025. PMC7025498 32116702

[25] Jiang H.-L. , Jin J.-Z. , and Wu D. , et al.Celastrol Exerts Synergistic Effects With PHA-665752 and Inhibits Tumor Growth of c-Met-Deficient Hepatocellular Carcinoma In Vivo, Molecular Biology Reports. (2013) 40, no. 7, 4203–4209, 10.1007/s11033-013-2501-y, 2-s2.0-84879411020. 23649759

[26] Xu Q. , Chen G. , and Xu H. , et al.Celastrol Attenuates RANKL-Induced Osteoclastogenesis In Vitro and Reduces Titanium Particle-Induced Osteolysis and Ovariectomy-Induced Bone Loss In Vivo, Frontiers in Pharmacology. (2021) 12, 10.3389/fphar.2021.682541, 682541. 34149427 PMC8210420

[27] Zeng D. , Zhang L. , and Luo Q. , Celastrol-Regulated Gut Microbiota and Bile Acid Metabolism Alleviate Hepatocellular Carcinoma Proliferation by Regulating the Interaction Between FXR and RXR*α* In Vivo and In Vitro, Frontiers in Pharmacology. (2023) 14, 10.3389/fphar.2023.1124240, 1124240. 36874033 PMC9975715

[28] Zhang B. , Zhong Q. , and Chen X. , et al.Neuroprotective Effects of Celastrol on Transient Global Cerebral Ischemia Rats via Regulating HMGB1/NF-*κ*B Signaling Pathway, Frontiers in Neuroscience. (2020) 14, 10.3389/fnins.2020.00847, 847. 32848589 PMC7433406

[29] Yu Y. , Wang J. , and Ruan L. , et al.Evaluation of Celastrol Antiviral Activity Against Equid Alphaherpesvirus Type 8 Infection, Viruses. (2025) 17, no. 3, 10.3390/v17030347, 347. 40143276 PMC11945448

[30] Yu J. S. , Tseng C. K. , and Lin C. K. , et al.Celastrol Inhibits Dengue Virus Replication via up-Regulating Type I Interferon and Downstream Interferon-Stimulated Responses, Antiviral Research. (2017) 57, 49–57, 10.1016/j.antiviral.2016.11.010, 2-s2.0-84995955836. PMC7113783 27847245

[31] Khalili N. , Karimi A. , Moradi M.-T. , and Shirzad H. , In Vitro Immunomodulatory Activity of Celastrol Against Influenza A Virus Infection, Immunopharmacology and Immunotoxicology. (2018) 40, no. 3, 250–255, 10.1080/08923973.2018.1440591, 2-s2.0-85042945462. 29493374

[32] Chen S.-R. , Li Z.-Q. , and Xu J. , et al.Celastrol Attenuates Hepatitis C Virus Translation and Inflammatory Response in Mice by Suppressing Heat Shock Protein 90*β* , Acta Pharmacologica Sinica. (2023) 44, no. 8, 1637–1648, 10.1038/s41401-023-01067-w. 36882503 PMC10374583

[33] Narayan V. , Ravindra K. C. , and Chiaro C. , et al.Celastrol Inhibits Tat-Mediated Human Immunodeficiency Virus (HIV) Transcription and Replication, Journal of Molecular Biology. (2011) 410, no. 5, 972–983, 10.1016/j.jmb.2011.04.013, 2-s2.0-79960351591. 21763500 PMC3140654

[34] Huang C.-L. , Chen D.-Y. , Tzang C.-C. , Lin J.-W. , Tzang B.-S. , and Hsu T.-C. , Celastrol Attenuates Human Parvovirus B19 NS1-Induced NLRP3 Inflammasome Activation in Macrophages, Molecular Medicine Reports. (2023) 28, no. 4, 10.3892/mmr.2023.13080. PMC10502933 37654202

[35] Chen G.-Y. , Pan Y.-C. , and Wu T.-Y. , et al.Potential Natural Products That Target the SARS-CoV-2 Spike Protein Identified by Structure-Based Virtual Screening, Isothermal Titration Calorimetry and Lentivirus Particles Pseudotyped (Vpp) Infection Assay, Journal of Traditional and Complementary Medicine. (2022) 12, no. 1, 73–89, 10.1016/j.jtcme.2021.09.002. 34549024 PMC8443859

[36] Fuzo C. A. , Martins R. B. , and Fraga-Silva T. , et al.Celastrol: A Lead Compound that Inhibits SARS-CoV-2 Replication, the Activity of Viral and Human Cysteine Proteases, and Virus-Induced IL-6 Secretion, Drug Development Research. (2022) 83, no. 7, 1623–1640, 10.1002/ddr.21982. 35989498 PMC9539158

[37] Que H. , Hong W. , and Lan T. , et al.Tripterin Liposome Relieves Severe Acute Respiratory Syndrome as a Potent COVID-19 Treatment, Signal Transduction and Targeted Therapy. (2022) 7, no. 1, 10.1038/s41392-022-01283-6, 399. 36566328 PMC9789731

[38] Yang F. , Jiang X. L. , and Tariq A. , et al.Potential Medicinal Plants Involved in Inhibiting 3CL(pro) Activity: A Practical Alternate Approach to Combating COVID-19, Journal of Integrative Medicine. (2022) 20, no. 6, 488–496, 10.1016/j.joim.2022.08.001. 35985974 PMC9359926

[39] Circu M. L. and Aw T. Y. , Reactive Oxygen Species, Cellular Redox Systems, and Apoptosis, Free Radical Biology and Medicine. (2010) 48, no. 6, 749–762, 10.1016/j.freeradbiomed.2009.12.022, 2-s2.0-76049083966. 20045723 PMC2823977

[40] Zhao F. , Ma X. , and Yang J. , et al.Investigation of Transmission and Evolution of PEDV Variants and Co-Infections in Northeast China From 2011 to 2022, Animals. (2024) 14, no. 15, 10.3390/ani14152168, 2168. 39123693 PMC11311072

[41] Arjin C. , Pringproa K. , and Hongsibsong S. , et al.In Vitro Screening Antiviral Activity of Thai Medicinal Plants Against Porcine Reproductive and Respiratory Syndrome Virus, BMC Veterinary Research. (2020) 16, no. 1, 10.1186/s12917-020-02320-8. PMC7106583 32228582

[42] Li S. , Xiao D. , and Zhao Y. , et al.Porcine Deltacoronavirus (PDCoV) Entry Into PK-15 Cells by Caveolae-Mediated Endocytosis, Viruses. (2022) 14, no. 3, 10.3390/v14030496, 496. 35336903 PMC8950576

[43] Xu Z. , Zhang Y. , and Cao Y. , The Roles of Apoptosis in Swine Response to Viral Infection and Pathogenesis of Swine Enteropathogenic Coronaviruses, Frontiers in Veterinary Science. (2020) 7, 10.3389/fvets.2020.572425, 572425. 33324698 PMC7725767

[44] Zhang X.-Z. , Tian W.-J. , Wang J. , You J.-L. , and Wang X.-J. , Death Receptor DR5 as a Proviral Factor for Viral Entry and Replication of Coronavirus PEDV, Viruses. (2022) 14, no. 12, 10.3390/v14122724, 2724. 36560727 PMC9783156

[45] Si F. , Hu X. , and Wang C. , et al.Porcine Epidemic Diarrhea Virus (PEDV) ORF3 Enhances Viral Proliferation by Inhibiting Apoptosis of Infected Cells, Viruses. (2020) 12, no. 2, 10.3390/v12020214, 214. 32075094 PMC7077256

[46] Xu X. , Ma M. , and Shi X. , et al.The Novel Nsp9-Interacting Host Factor H2BE Promotes PEDV Replication by Inhibiting Endoplasmic Reticulum Stress-Mediated Apoptosis, Veterinary Research. (2023) 54, no. 1, 10.1186/s13567-023-01158-w, 27. 36949543 PMC10035214

[47] Wang X. , Liu Y. , Li K. , Yang M. , Wang Q. , and Hao Z. , Triacetyl Resveratrol Inhibits PEDV by Inducing the Early Apoptosis In Vitro, International Journal of Molecular Sciences. (2022) 23, no. 23, 10.3390/ijms232314499, 14499. 36498827 PMC9737061

[48] An X. , Yu W. , Liu J. , Tang D. , Yang L. , and Chen X. , Oxidative Cell Death in Cancer: Mechanisms and Therapeutic Opportunities, Cell Death & Disease. (2024) 15, no. 8, 10.1038/s41419-024-06939-5, 556. 39090114 PMC11294602

[49] Chen X. , Zhao Y. , and Luo W. , et al.Celastrol Induces ROS-Mediated Apoptosis via Directly Targeting Peroxiredoxin-2 in Gastric Cancer Cells, Theranostics. (2020) 10, no. 22, 10290–10308, 10.7150/thno.46728. 32929349 PMC7481428

[50] Zhou Y. , Zhang Y. , and Dong W. , et al.Porcine Epidemic Diarrhea Virus Activates PERK-ROS Axis to Benefit its Replication in Vero E6 Cells, Veterinary Research. (2023) 54, no. 1, 10.1186/s13567-023-01139-z, 9. 36737830 PMC9897154

[51] Hu M. , Bogoyevitch M. A. , and Jans D. A. , Subversion of Host Cell Mitochondria by RSV to Favor Virus Production is Dependent on Inhibition of Mitochondrial Complex I and ROS Generation, Cells. (2019) 8, no. 11, 10.3390/cells8111417, 1417. 31717900 PMC6912631

[52] Tu Y. , Dai G. , Chen Y. , Tan L. , Liu H. , and Chen M. , Emerging Target Discovery Strategies Drive the Decoding of Therapeutic Power of Natural Products and Further Drug Development: A Case Study of Celastrol, Exploration. (2025) 5, no. 4, 10.1002/EXP.20240247, e20240247. 40873643 PMC12380071

[53] Chen S. , Wang Z. , and Gao J. , et al.Rapid Discovery of Celastrol Derivatives as Potent and Selective PRDX1 Inhibitors via Microplate-Based Parallel Compound Library and In Situ Screening, Journal of Medicinal Chemistry. (2025) 68, no. 13, 13609–13627, 10.1021/acs.jmedchem.5c00433. 40546088

[54] Meng Y. , Jiang X. , Raj R. , Shen P. , and Zhang J. , Exploring the Molecular Interaction of Celastrol and HMGB1 by Multi-Spectra Analysis, Journal of Biomolecular Structure and Dynamics. (2025) 1–15, 10.1080/07391102.2025.2530041. 40702940

[55] Kamel E. M. , Ali M. , Allam A. A. , Ahmed N. A. , Aba A. F. , and Lamsabhi A. M. , Disrupting the Hsp90-Cdc37 Axis: A Selective Strategy for Targeting Oncogenic Kinases in Cancer, RSC Advances. (2025) 15, no. 24, 19376–19391, 10.1039/D5RA03137K. 40491798 PMC12147015

[56] Xiang S. , Chen J. , and Deng M. , et al.Celastrol Ameliorates Experimental Autoimmune Uveitis Through STAT3 Targeting and Gut Microenvironment Reprofiling, International Immunopharmacology. (2024) 127, no. doi, 10.1016/j.intimp.2023.111339, 111339. 38064813

[57] Luo P. , Zhang Q. , and Zhong T.-Y. , et al.Celastrol Mitigates Inflammation in Sepsis by Inhibiting the PKM2-Dependent Warburg Effect, Military Medical Research. (2022) 9, no. 1, 10.1186/s40779-022-00381-4, 20. 35596191 PMC9121578

[58] Schiavone S. , Morgese M. G. , Tucci P. , and Trabace L. , The Therapeutic Potential of Celastrol in Central Nervous System Disorders: Highlights From In Vitro and In Vivo Approaches, Molecules. (2021) 26, no. 15, 10.3390/molecules26154700, 4700. 34361850 PMC8347599

[59] Xu L.-N. , Zhao N. , and Chen J.-Y. , et al.Celastrol Inhibits the Growth of Ovarian Cancer Cells In Vitro and In Vivo, Frontiers in Oncology. (2019) 9, no. doi, 10.3389/fonc.2019.00002, 2-s2.0-85063297534, 2. 30746340 PMC6360154

