# Supplementary material for: Celastrol Inhibits Porcine Epidemic Diarrhea Virus Replication by Promoting ROS‐Mediated Apoptosis
Source: Transbound Emerg Dis. 2025 Dec 12;2025:4020619. doi: 10.1155/tbed/4020619 (PMC12699546; doi:10.1155/tbed/4020619)
Supplement: Supplementary file 1 — Supporting Information Figure S1: Binding conformations of celastrol bound to the top 10 hub proteins generated by virtual ligand docking. Figure S2: Time‐dependent effects of celastrol on ROS and apoptosis in PEDV‐Infected Cells. Figure S3: Co‐incubation with Z‐FAM‐VAD failed to impact celastrol’s antiviral efficacy against PEDV. [file TBED-2025-4020619-s001.docx]

**Supporting Information**

Supplementary Fig S1


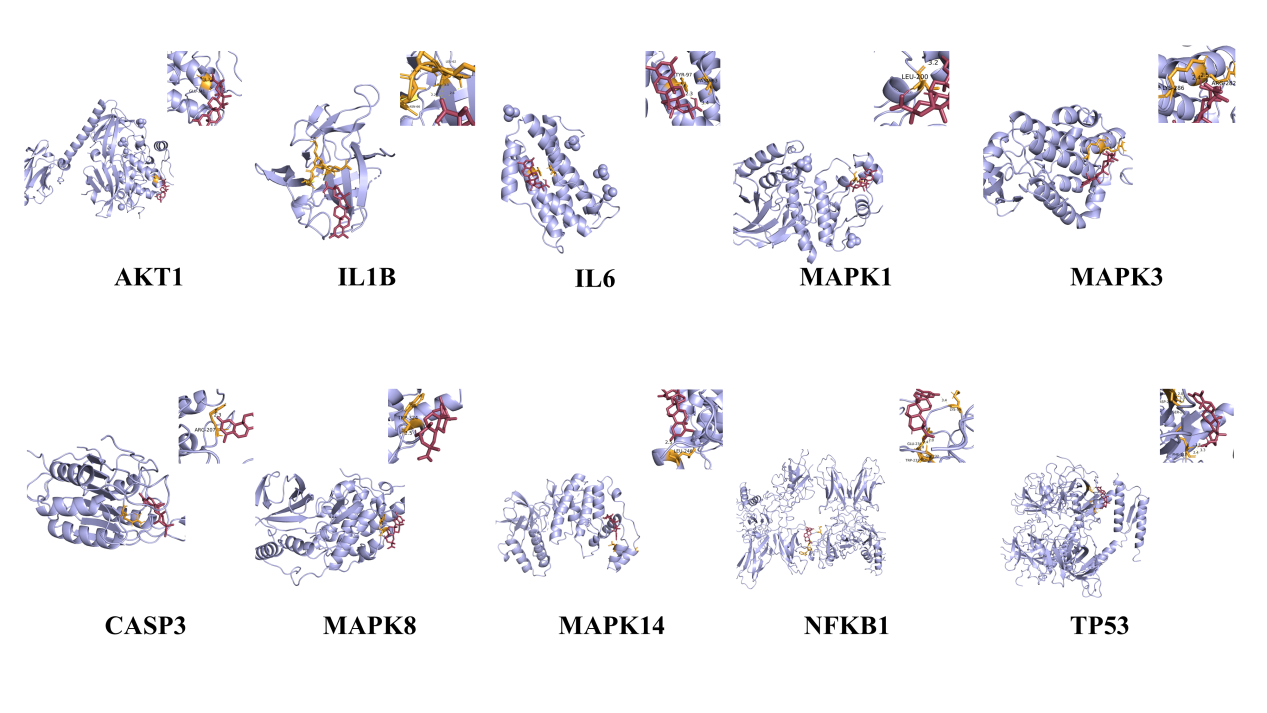


**Supplementary FIG S1** Binding conformations of celastrol bound to the top 10 hub proteins generated by virtual ligand docking.

Supplementary Fig S2


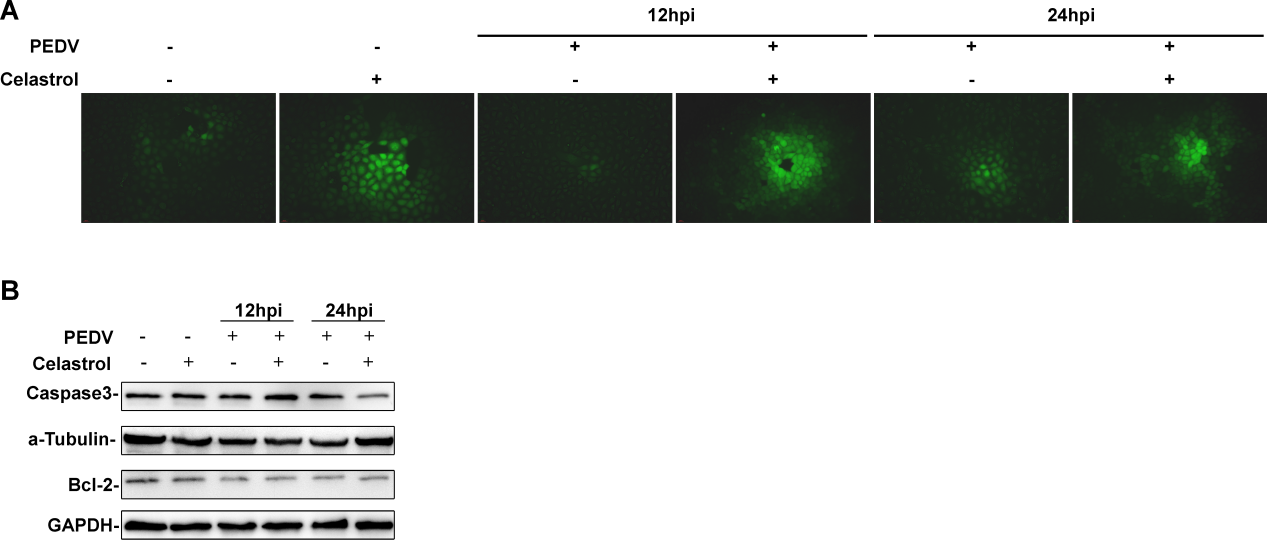


**Supplementary FIG S2** Time-dependent effects of celastrol on ROS and apoptosis in PEDV-Infected Cells. (A) Intracellular ROS levels were detected by DCF fluorescence intensity, scale bar = 20 μM. (B) Western blotting analysis of apoptosis marker in PEDV-infected Vero-E6 cells treated with celastrol at 12 and 24 hpi.

Supplementary Fig S3


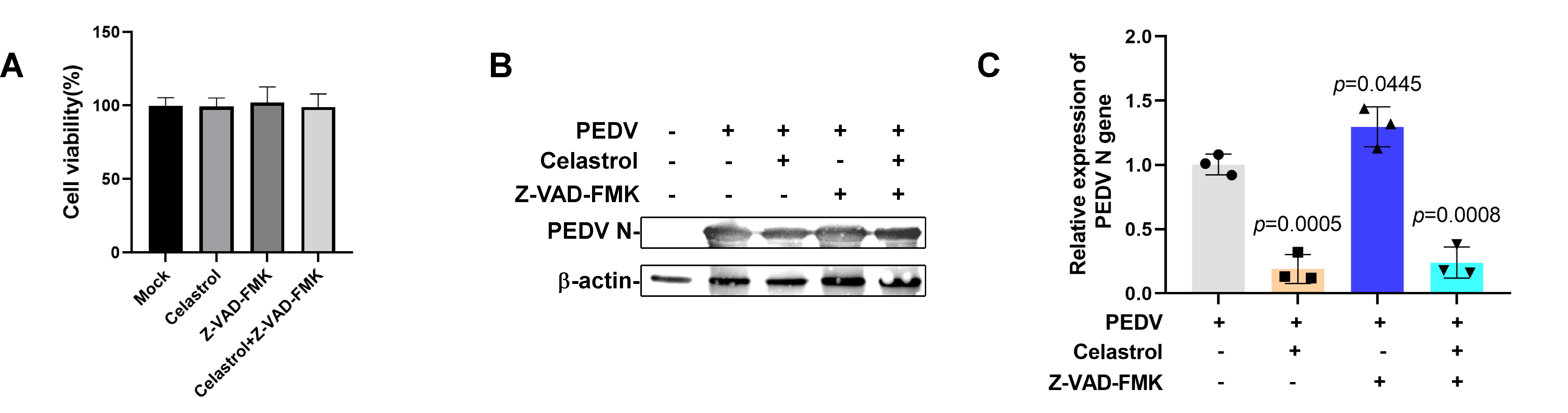


**Supplementary FIG S3** Co-incubation with Z-FAM-VAD failed to impact celastrol's antiviral efficacy against PEDV. (A) Cytoxicity of Z-VAD-FMK in Vero-E6 cells at 24 hpi using MTT assay. (B) PEDV N protein level in Vero-E6 cells under celastrol and Z-VAD-FMK treatment was detected by Western blotting assay. (C) PEDV N gene level in Vero-E6 cells under celastrol and Z-VAD-FMK treatment was detected by RT-qPCR. Each datum represents the results of three independent experiments (mean ± SD, n = 3). The data were compared using Student’s *t*-tests.
